# Anisotropic hyperelastic properties of porcine pericardium under equibiaxial loading: implications for aortic valve design

**DOI:** 10.3389/fbioe.2026.1840657

**Published:** 2026-08-26

**Authors:** Edward Matjeka, Nikita Pil, Alex G. Kuchumov, Harry M. Ngwangwa, Thanyani Pandelani, Fulufhelo Nemavhola

**Affiliations:** 1 Department of Mechanical, Bioresources and Biomedical Engineering, School of Engineering, College of Science, Engineering and Technology, University of South Africa, Pretoria, South Africa; 2 Research Center for Genetics and Life Sciences, Sirius University of Science and Technology, Sirius, Russia; 3 Biofluids Laboratory, Perm National Research Polytechnic University, Perm, Russia; 4 Department of Mechanical Engineering, Faculty of Engineering and the Built Environment, Durban University of Technology, Durban, South Africa

**Keywords:** aortic valve, biaxial test, fluid–structure interaction, prosthesis, simulation

## Abstract

**Background and objective:**

Bioprosthetic aortic valves with leaflets made from porcine-derived soft tissue are used to treat aortic valve disease. To improve the durability and hemodynamic performance of bioprosthetic aortic valves, it is important to understand the biomechanics of these tissues and to assess the hemodynamic performance by quantifying orifice area, downstream jet metrics, and flow parameters. In this study, we aim to investigate the mechanical properties of porcine pericardium, derive an appropriate constitutive model, and apply it in fluid–structure interaction (FSI) simulations to evaluate the hemodynamic performance of a bioprosthetic aortic valve device.

**Methods:**

Porcine pericardium was characterized using equibiaxial tensile testing. Immediately after the slaughter of 51-week-old pigs, nine hearts with intact pericardium were carefully dissected. After performing equibiaxial tensile tests, the anisotropic hyperelastic behavior of porcine pericardium was characterized; ten anisotropic constitutive models were then used to identify the material parameters. Subsequently, these parameters were used in FSI computations of a bioprosthetic valve.

**Results:**

Using an evaluation index that considered only the coefficient of determination, it was found that the Fung model, four-fiber-family model, and HGO model fitted the data better than the other models. For the FSI simulations, the Holzapfel (2005) model and the four-fiber-family models were selected because their explicitly fiber-reinforced formulations allow the constitutive description to be directly linked to the prescribed leaflet fiber architecture. The selected models were then used for FSI modeling of aortic valve hemodynamics, and the computed results were consistent with literature-based numerical data. The peak velocity was 1.77 m/s at 323 ms for the Holzapfel (2005) model and 1.94 m/s at 315 ms for the four-fiber-family model. The difference was 0.17 m/s, corresponding to 9%, while the waveform shapes remained qualitatively consistent across models, with a rapid systolic increase followed by a gradual decay during ejection. The obtained values fall within the range expected for normal aortic valve function.

**Conclusion:**

To fully characterize the mechanical response of porcine pericardium and identify the most predictive constitutive model, it is recommended to combine uniaxial tension, shear, and biaxial tests rather than relying on a single loading mode.

## 1 Introduction

The leading causes of death worldwide are cardiovascular events, and approximately 50% of cardiovascular deaths are due to aortic valve failure (Faure et al., 2026). Two common aortic valve failures are stenosis and regurgitation (Gupta et al., 2025). The treatment of dysfunctional aortic valves is performed by replacing the native aortic valve with bioprosthetic or mechanical aortic valves through surgical or transcatheter interventions (Liu et al., 2025). The downsides of mechanical and bioprosthetic valves are impaired hemodynamics and inferior durability, respectively (Tillquist and Maddox, 2011; Head et al., 2017). Computational fluid dynamics (CFD) and fluid–structure interaction (FSI) approaches can contribute to analyzing the hemodynamic and structural performance of cardiovascular devices (Kuchumov A. et al., 2020; Kamaltdinov and Kuchumov, 2021; Khairulin et al., 2024) and other applications (Kuchumov, 2018; 2019; Kuchumov A. G. et al., 2020). FSI computations can be used to evaluate not only healthy-state hemodynamics (Pil et al., 2023) or pathology (Fedotova et al., 2025) but also treatment methods such as TAVI (Mutlu et al., 2024).

Bioprosthetic aortic valves made from porcine soft tissues are derived from aortic valve leaflets and the pericardium (Grunkemeier et al., 2012) (Huang et al., 2025), and continued efforts are underway to improve the durability and hemodynamic performance of bioprosthetic aortic valves in general (Chen et al., 2025).

To enhance the lifespan and hemodynamic performance of soft tissues, it is important to understand the biomechanics of these tissues (Pandelani et al., 2025). Porcine pericardium has been used in developing bioprosthetic aortic valves, although there is a need for better understanding of the mechanical properties of this soft tissue (Campion et al., 2021). The progression of diseases can be better understood by experimental tissue testing, which can serve as a useful tool for clinicians (Nemavhola et al., 2021a).

Finite element models have been developed to predict the response of the porcine pericardium when subjected to different loading conditions (Rassoli et al., 2020; 2022). The challenges associated with the use of these constitutive models are that some of them neglect certain aspects of tissue physics (Nemavhola et al., 2022; Pandelani et al., 2025). Currently, some models are physics-oriented (Ndlovu et al., 2022). To our knowledge, few studies have reported their accuracy in predicting the response of porcine pericardium when subjected to biaxial tensile tests. Accurate material parameters derived from accurate constitutive models can lead to accurate predictions of the lifespan and hemodynamics of the bioprosthetic aortic valve made of porcine pericardium (Stefanie et al., 2015). The constitutive models used in this study are physics-based and are as follows: Holzapfel (2000), Holzapfel (2005), Holzapfel and Ogden (2015), Gasser et al. (2006), polynomial (anisotropic), Choi–Vito, Fung anisotropic, transversely isotropic, and anisotropic 8-chain hyperelastic models.

The hemodynamic performance of tissue-based bioprosthetic aortic valves is commonly assessed through a combination of global and local indicators that reflect both opening efficiency and the near-wall flow environment (Smadi et al., 2020; Scarpolini et al., 2025). Global metrics such as the geometric orifice area (GOA) and effective orifice area (EOA) quantify the instantaneous anatomical opening and the minimal downstream jet cross-section that governs energy loss, with the effective orifice area typically being smaller and more sensitive to inflow shape and root compliance (El-Nashar et al., 2024). Local metrics derived from velocity gradients, including wall shear stress (WSS) and its directional variability, provide a mechanistic bridge to adverse biological responses because altered shear patterns are associated with vascular and valvular remodeling and can contribute to degeneration processes in the long term (Yin et al., 2025). Costa et al. (2025a) have shown that WSS-based parameters are important indicators of long-term degradation in tissue-based bioprosthetic valves since leaflet flutter can alter near-wall shear distributions, flow structures in the Valsalva sinuses, and descriptors associated with calcification and endothelial activation. Multiscale analyses of failed bioprosthetic valves further demonstrated that mineralization and collagen network alterations spatially coincide with regions of elevated hemodynamic and biomechanical indicators, including time-averaged wall shear stress (TAWSS), oscillatory shear index (OSI), relative residence time (RRT), and topological shear variation index (TSVI), supporting the use of WSS-derived metrics to identify calcification-prone and degeneration-prone leaflet regions (Tsolaki et al., 2023).

Structural valve deterioration may manifest as thickening, calcification, or tearing, leading to functional stenosis or regurgitation and altered transvalvular flow patterns (Fazzari et al., 2023; Yin et al., 2025). In this context, disturbed flow features and asymmetric jets may drive spatially heterogeneous shear environments that are not fully captured by routine clinical measurements. The ability to resolve shear-based indices such as the OSI and RRT is particularly valuable because elevated values are linked to increased residence time and oscillation near surfaces, conditions that are often discussed in relation to thrombus prone environments (Moon et al., 2025; Yang J. et al., 2025).

Computational hemodynamics of tissue-based bioprosthetic aortic valves has evolved from simplified, decoupled strategies. In these earlier approaches, flow is computed in a fixed geometry or with prescribed leaflet kinematics, while leaflet mechanics are solved separately under an imposed pressure load (Bailoor et al., 2021). This enables consistent evaluation of both global opening metrics, such as EOA and GOA, and local shear-related quantities such as WSS and indices of shear unsteadiness including OSI, which are particularly relevant when studying stenosis- and calcification-associated disturbed flow patterns (Fedotova et al., 2025; Pil et al., 2026). A practical advantage in this setting is provided by the arbitrary Lagrangian–Eulerian (ALE) moving-mesh formulation because a boundary-conforming interface and preserved near-wall resolution improve the reliability of WSS and derivative metrics that are highly sensitive to near-wall velocity gradients (Costa et al., 2025b).

Comparative studies further indicate that the coupling strategy can materially affect predicted leaflet dynamics; for leaflet flutter, FSI better reproduces the experimental behavior, and a reported comparison showed an error of approximately 5% in peak GOA for FSI *versus* 46.5% for a structural FEM-only approach, highlighting the risk of misleading conclusions regarding cyclic stresses and fatigue durability when fluid feedback is neglected (Costa et al., 2025b). Similarly, closure dynamics depend on jet structure and pressure distribution, with bioprosthetic valves exhibiting an earlier tendency to close during systolic deceleration under a dominant central jet, which emphasizes the need to model the full cardiac cycle rather than peak opening alone (Carrel et al., 2023).

The aim of the present study is to comparatively assess the suitability of 10 anisotropic hyperelastic constitutive models for describing the mechanical response of native porcine pericardium based on equibiaxial testing data and to subsequently apply the selected models in FSI simulations of bioprosthetic aortic valve hemodynamics. Rather than merely confirming the already known anisotropic behavior of porcine pericardium, we evaluate which available constitutive formulations most accurately reproduce the experimental data and how differences in leaflet material description affect predicted valve performance, including leaflet kinematics, flow velocity, pressure gradients, orifice area, and WSS.

The study hypothesis is that the choice of an anisotropic hyperelastic constitutive model identified from equibiaxial testing of porcine pericardium affects not only the quality of fitting the experimental mechanical response of the tissue but also the predicted kinematic and hemodynamic characteristics of a bioprosthetic aortic valve in an FSI model. To test this hypothesis, 10 anisotropic hyperelastic constitutive models were fitted to the experimental data, and the best-performing models were selected using the evaluation index proposed by Nemavhola et al. (2021a) and Nemavhola et al. (2021b).

The manuscript is arranged as follows. Section 2 describes the biaxial test procedure and the constitutive models adopted in the study. Optimization algorithms for parameter estimation are introduced. The FSI model of an aortic valve bioprosthesis with porcine leaflets described by selected constitutive relations is considered. Additionally, this section contains information about the mesh, material models, and FSI settings. Section 3 presents the results of experimental studies and simulations. Section 4 contains the obtained results, which are discussed and compared with those of other studies. The study’s limitations are also considered in Section 4.3.

## 2 Methods

We propose a comprehensive study on the mechanical properties of porcine pericardium. The first part of the study involved conducting full-scale biaxial tensile tests of samples. Next, parameters were selected for 10 hyperelastic material models. Hyperelastic constitutive models must be formulated in terms of finite (true) strain measures, typically the Green–Lagrange strain tensor and the second Piola–Kirchhoff stress tensor, or the Cauchy stress with appropriate strain invariants. Our use of engineering stress and strain in the initial data processing section was solely for the purpose of presenting the raw experimental data and performing the initial parameter optimization. Engineering measures were not directly used as inputs to the hyperelastic models. Although engineering measures are convenient for experimental reporting, the subsequent conversion to finite strain measures introduces some sensitivity to the assumed deformation field homogeneity, which is a limitation of the biaxial testing setup. Nevertheless, it should be noticed that the resultant strain was less than 10%, so we found that the difference between the engineering and true strains was negligible. The models were also evaluated based on the evaluation index. The second part of the study involved the application of the obtained material models in numerical experiments. Based on the evaluation index, the optimal models were applied to simulate the hemodynamics of the aortic valve. In the next step, the models that showed the best agreement with the experimental data were applied in coupled FSI simulations of a bioprosthetic aortic valve to evaluate the influence of leaflet constitutive description on hemodynamic and mechanical performance.

### 2.1 Biaxial tensile tests

#### 2.1.1 Material acquisition

Animal studies were approved by the University of South Africa, Pretoria, South Africa, and by the Ethics Committee of the College of Agriculture and Environmental Sciences, Animal REC, on 09 January 2025. The experiments were conducted in accordance with local legislation and institutional requirements.

**FIGURE 1:**
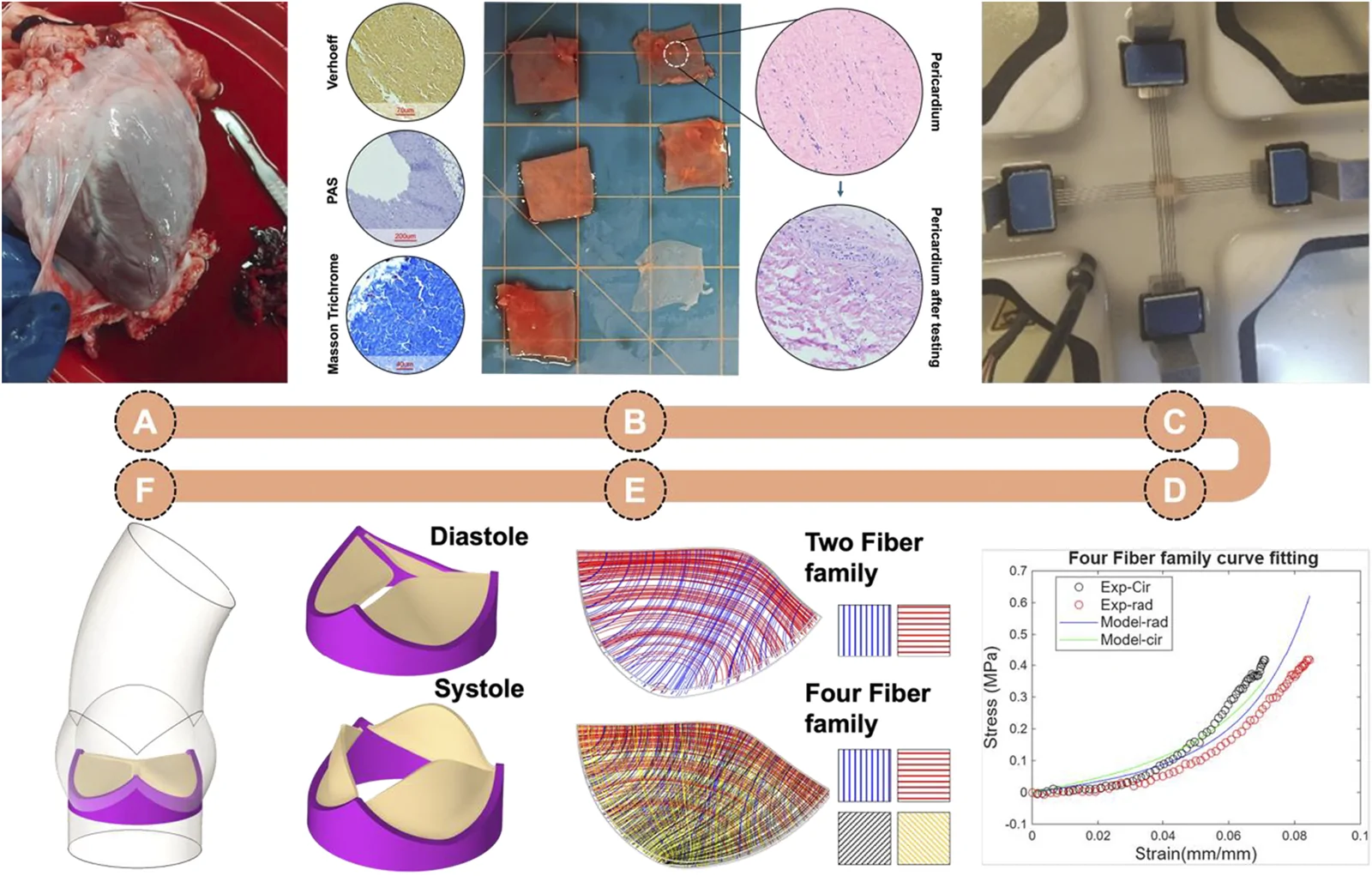
Design of the study. **(A)** Porcine heart with pericardium, **(B)** tissue samples with fiber orientation, **(C)** biaxial tensile tester CellScale, **(D)** fitting curve from biaxial testing, **(E)** aortic valve leaflet with 2- and 4-fibers family, and **(F)** finite element model for FSI simulation.

Native porcine pericardium was used for biaxial tensile characterization. Fresh hearts from 51-week-old Landrace pigs weighing 100–110 kg were obtained from a local abattoir and transported immediately after slaughter to the biomedical engineering laboratory in an ice cooler box. Nine hearts were dissected with the pericardium preserved. The pericardium was removed from each heart, and square specimens with dimensions of 10 × 10 mm^2^ were cut using scalpels and a measurement-sized cutting board. The average specimen thickness was 0.2 mm. Thickness was measured using an adapted Vernier caliper to ensure consistent measurements while minimizing tissue compression. Before testing, the circumferential and axial directions were marked to preserve the anatomical orientation of each specimen (Inoue et al., 2021; Donmazov et al., 2024). Figure 1 shows a porcine heart with the pericardium.

#### 2.1.2 Biaxial testing

Biaxial tensile tests were performed using a CellScale Biotester. The specimens were mounted in a testing chamber filled with phosphate-buffered saline (PBS) to maintain tissue hydration. The temperature was increased and maintained at 37 °C under approximate physiological testing conditions (Nemavhola, 2021). Each specimen was fixed using four bio-rakes mounted on four goosenecks, with five tines on each bio-rake. The tine diameter was 305 μm, the tine spacing was 1 mm, and the puncture depth was 1.9 mm. The specimen was transferred to the biotester using a specimen holding plate, which facilitated reproducible mounting and reduced handling-induced deformation. The specimen was aligned so that the circumferential and axial material directions corresponded to the two loading axes of the testing system. A sample cut to size was taken to the biotester using a specimen holding plate, which facilitated mounting the specimen onto the biotester. The gauge region was located in the central part of the gripped specimen and had an approximate area of 8 × 8 mm^2^. To run the tests, the testing station is lowered to allow access to the fluid chamber, and PBS was added. The fluid chamber was then placed back into position, and the bio-rakes were mounted onto four goosenecks.

Equibiaxial loading was applied in a force-controlled mode with a maximum force of 1,000 mN. The force-controlled protocol was selected to reproduce tensile loading conditions relevant to the physiological loading of aortic valve tissues. Before the main tensile test, specimens were preconditioned by applying a preload of 25 mN, followed by incremental loading to 10% of the maximum force over 20 s. This was followed by a 3-s hold at 10% of the maximum force, a 20-s recovery period, and a 3-s rest. This sequence was repeated for 10 preconditioning cycles before specimen testing.

For the main stretch test, the specimens were loaded to a maximum force of 1,000 mN over 20 s, followed by a 20-s recovery period. Each test was performed three times, and the data from the third cycle were used as the experimental results. After testing, the specimens were unmounted and discarded. The selected strain rate was 0.005 s^−1^, which was chosen to approximate physiological quasi-static loading conditions. The nominal force of the load cell was 23 N, and its resolution was 10 mN.

**FIGURE 2:**
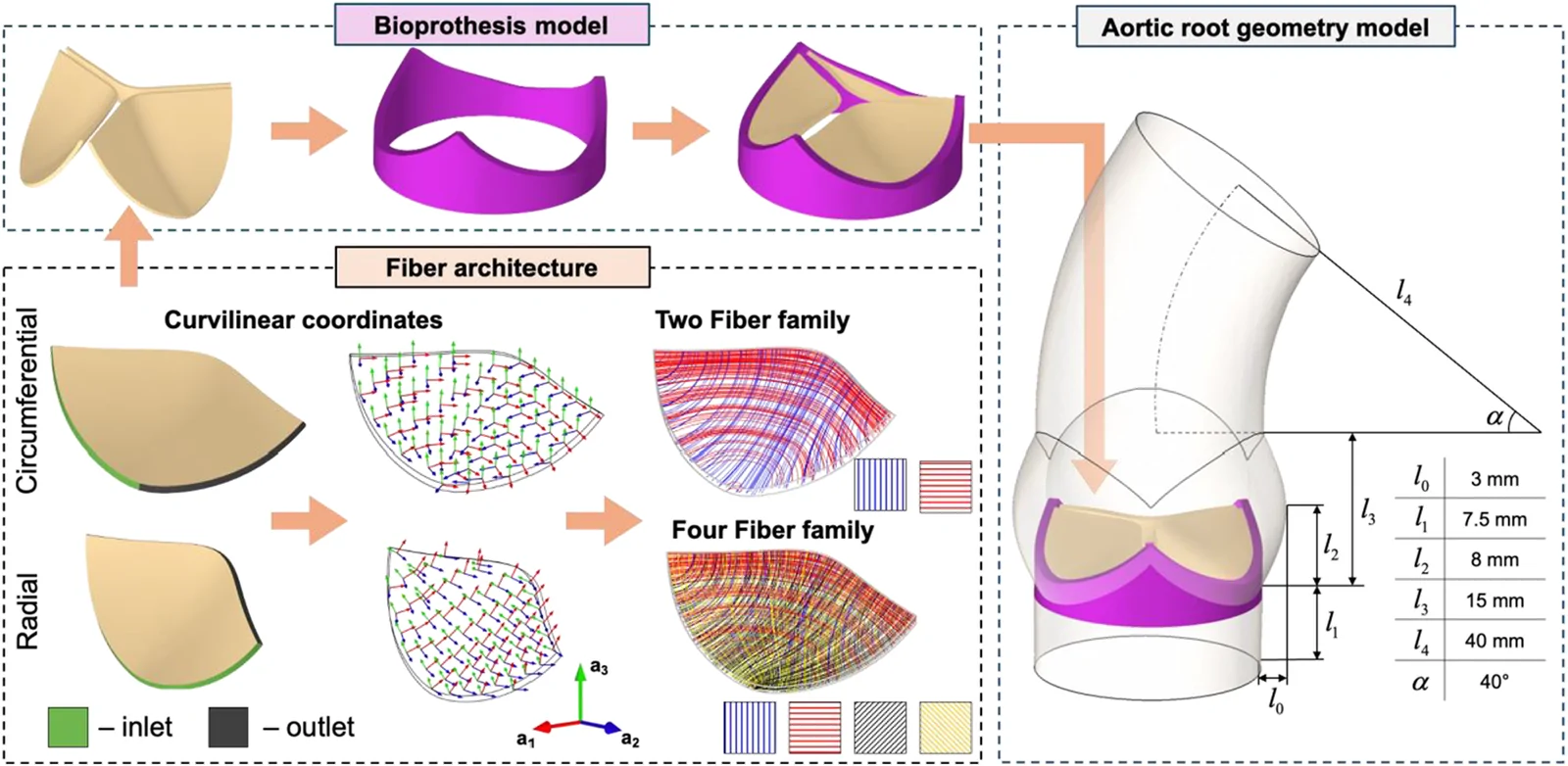
Scheme of curvilinear coordinates and fiber’s architecture.

The obtained stress–strain curves were used to characterize the anisotropic mechanical response of porcine pericardium. The cross-sectional area was calculated as the product of the initial specimen width and the average measured thickness. The experimental responses in the circumferential and axial directions were processed separately. First, the stress–strain curves obtained from individual specimens were used to identify the material parameters of 10 anisotropic hyperelastic constitutive models. Then, the average parameters obtained for each model were used to reproduce independent averaged experimental responses. This procedure allowed the fitting quality for individual tests and the predictive capability of each constitutive model for averaged biaxial tissue behavior to be assessed. The quality of model fitting was evaluated using the coefficient of determination and the evaluation index. The models that showed the best agreement with the biaxial experimental data were then selected for further use in the FSI simulations of the bioprosthetic aortic valve.

### 2.2 Constitutive models

The recorded data are displayed as force and deformation, and Equations 1–3 were used to transfer the data to stress and strain.

$$A=L_{0}\times t,$$ (1)

$$\sigma_{i}=\frac{F_{i}}{A_{i}},$$ (2)

$$\varepsilon_{i}=\frac{l_{i}-l_{0,i}}{l_{0,i}},$$ (3)

where ε is the engineering strain, $l_{0,i}$ and $l_{i}$ are the initial and current lengths in the corresponding loading direction, respectively, σ is the engineering stress, t is the thickness of the material, $F_{i}$ is the measured force in the i -direction, and $A_{i}$ is the initial cross-sectional area of the specimen in the corresponding direction.

Many constitutive models for characterizing soft tissues have been derived; in this study, 10 constitutive models have been used to help characterize porcine pericardium. Since porcine pericardium is an anisotropic hyperelastic material, 10 constitutive models were used to identify optimized material parameters: Fung, described by Zhang and Kassab et al. (2007) and Murphy and Rajagopal et al. (2021); Choi–Vito, described by Kural et al. (2012) and Ngwangwa et al. (2022); Polynomial anisotropic, described by Ndlovu et al. (2022); Holzapfel-2000, described by Holzapfel (2000) and Zulliger et al. (2004); Holzapfel-2005, described by Holzapfel et al. (2005), Holzapfel (2006) and Holzapfel and Ogden et al. (2010); Four-fiber family, described by Di Achille et al. (2011), Li et al. (2014), Li et al. (2015), and Caulk et al. (2015); Gasser et al. (2006); Holzapfel and Ogden (2015); Transversely Isotropic, described by Anssari-Benam et al. (2018); and Anisotropic 8-chain, described by Yang et al. (2025b). The detailed formulations of these constitutive models are presented in Supplementary Material S1.

### 2.3 Optimization methods

The Levenberg–Marquardt algorithm (Gavin, 2024) and the genetic algorithm were used to obtain parameters of constitutive relations. The metrics used to select an optimized constitutive model with optimum parameters include the sum of squares of errors, the sum of absolute errors, normalized errors, normalized root mean squared errors (Hodson, 2022), the coefficient of correlation, and the coefficient of determination (Renaud and Victoria-Feser, 2010; Chicco et al., 2021). The details are presented in Supplementary Material S2.

### 2.4 Fluid–structure interactions aortic valve model

Two-way FSI analysis of a bioprosthesis valve in the aorta segment was performed. Blood flow is considered an incompressible Newtonian fluid flow, with a constant density of 1,060 kg/m^−3^ and dynamic viscosity of 0.004 Pa∙s. The average Reynolds number is 3,685 and therefore lies within the transitional range. The flow was considered laminar; this assumption was adopted to comparatively assess the influence of leaflet constitutive models under identical boundary conditions and numerical settings.

The Navier–Stokes equation and the continuity equation (Equations 4–6) were used to describe the fluid flow:

$$\nabla \cdot \upsilon_{f}=0,$$ (4)

$$\rho \frac{\partial \upsilon_{f}}{\partial t}+\rho \left(\upsilon_{f}\cdot \nabla \right)\upsilon_{f}=\nabla \cdot \sigma_{f},$$ (5)

$$\sigma_{f}=-pI+\tau ,$$ (6)

where $\upsilon_{f}$ is the fluid velocity, $\sigma_{f}$ is the stress tensor, ρ is the fluid density, p is the pressure, and τ is the viscous stress tensor.

The pressure is determined at the outlet boundary using a two-element Windkessel model (Equations 7–9):

$$C\frac{dP_{Wk}}{dt}+\frac{P_{Wk}}{R_{p}}=Q_{Ao},$$ (7)

$$P_{Ao}\left(t\right)=Q_{Ao}R_{c}+P_{Wk},$$ (8)

$$p_{out}=P_{Ao}\left(t\right),$$ (9)

where $Q_{Ao}$ is the flow rate in the aorta, mL·s^−1^; $R_{c}$ is the characteristic resistance, mmHg∙mL^−1^∙s; $R_{p}$ is the peripheral resistance, mmHg∙mL^−1^∙s; and C is arterial compliance, mm Hg^−−1^∙mL. $P_{Ao}\left(t\right)$ and $P_{Wk}\left(t\right)$ represent the aortic pressure and pressure stored within the Windkessel model at time *t*, respectively. The parameters of the Windkessel model were tuned to provide physiologically consistent pressure, flow-rate, and transvalvular pressure-gradient values during the simulated cardiac cycle.

This model represents an idealized bioprosthetic aortic valve with parameterized leaflet geometry, which was previously described (Pil and Kuchumov, 2024). The bioprosthetic frame was rigidly fixed within the aortic root, and the aortic root wall was treated as a fixed boundary. Therefore, the effect of surrounding aortic tissues was not explicitly included in the present FSI model. This modeling choice was used to provide a controlled computational setup and to focus on the influence of leaflet constitutive behavior on valve kinematics and hemodynamic indices.

The FSI domain comprises a tricuspid aortic valve coupled to an aortic root and the proximal ascending aorta segment. The geometry used in this study is idealized and parameterized. The aortic root and leaflet dimensions were selected from commonly reported literature values for a normal adult aortic root (Patel et al., 2022; Yin et al., 2024) and are summarized in Figure 2. The model includes three sinuses of Valsalva, a sinotubular junction, and a curved ascending aorta segment to reproduce the eccentric jet development observed in the previous *in silico* studies. The three leaflets were generated as identical surfaces attached to the annulus and commissures. This idealized setup was chosen to isolate the effect of leaflet-constitutive assumptions on the predicted valve kinematics and hemodynamic metrics.

**FIGURE 3:**
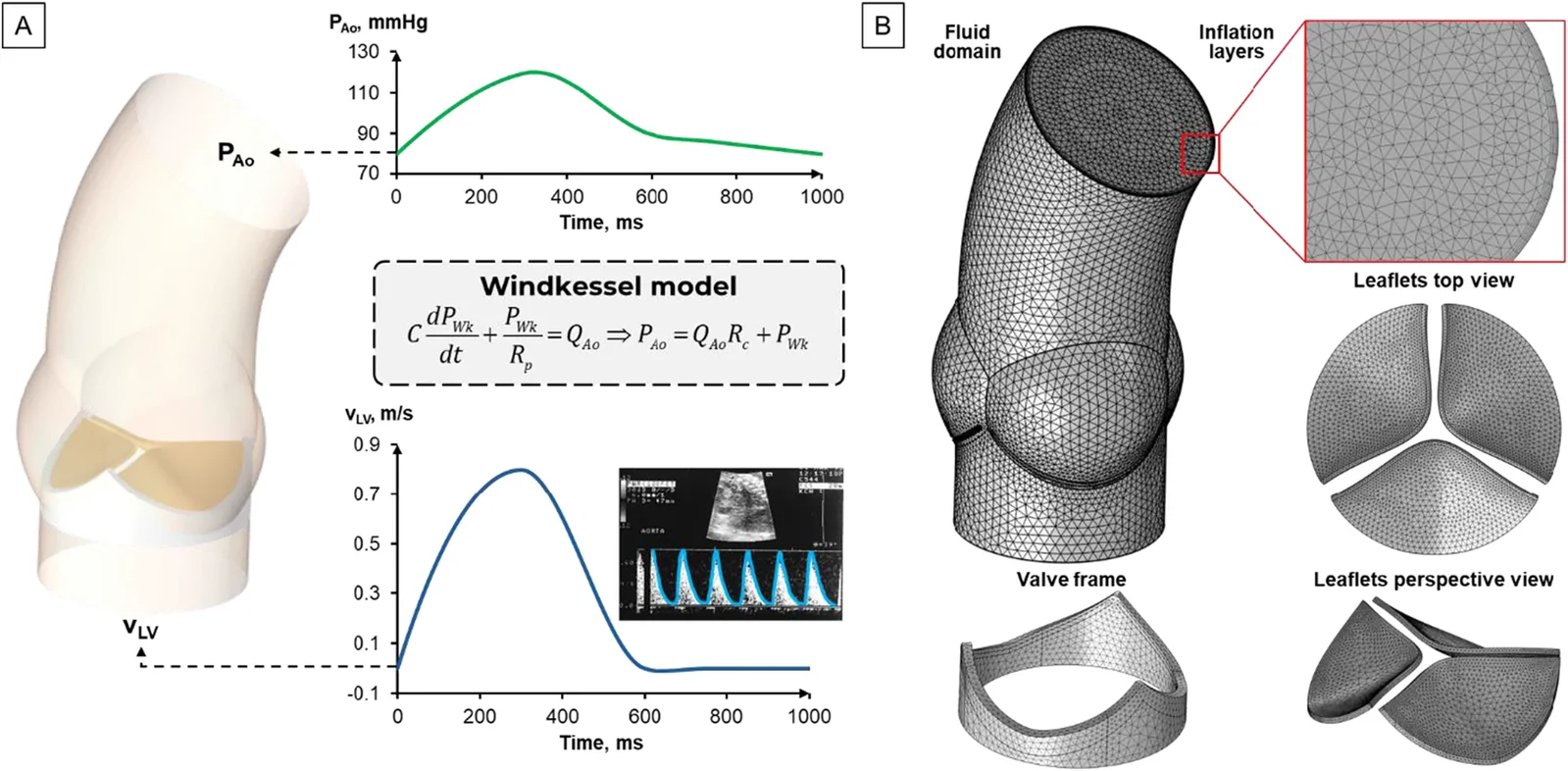
**(a)** – inlet and outlet boundary conditions, **(b)** – finite element mesh

The first-order differential equation was solved numerically using the fourth-order Runge–Kutta solution. At the current time step, we computed $P_{Ao}\left(t\right)$ using the value of $P_{Wk}\left(t\right)$ defined by the Runge–Kutta solution at the same time step. The model parameters are C=3.128 mmHg^−1^∙mL, $R_{p}=$ 0.6652 mmHg∙mL^−1^∙s, and $R_{c}$ = 0.0914 mmHg mL^−1^∙s. The values for diastolic and systolic pressures are taken as 80 mmHg and 120 mmHg, respectively (Figure 3A).

The anisotropic hyperelastic behavior of aortic valve leaflets are shown in Equations 10, 11:

$$\rho \frac{\partial^{2}u_{s}}{\partial t^{2}}=\nabla \cdot \sigma_{s},$$ (10)

$$\sigma_{s}=\frac{\partial W}{\partial \varepsilon },$$ (11)

where ρ is the density, $u_{s}$ is the displacement field, and $\sigma_{s}$ is the Cauchy stress tensor.

The anisotropic hyperelastic constitutive models were applied to the deformable valve leaflets, which represent porcine pericardium in the bioprosthetic aortic valve model. The aortic root and proximal ascending aorta were treated as rigid walls; therefore, no constitutive model was assigned to the aortic wall. The governing equations for the deformable leaflet domain are presented in Equations 12, 13:

$$W_{1}=\mu \left(I_{1}-3\right)+\sum_{i=1}^{2}\frac{k_{1}}{2k_{2}}\left\{e^{k_{2}\left[\left(1-\rho \right){\left(I_{1}-3\right)}^{2}+\rho {\left(I_{4i}-1\right)}^{2}\right]}-1\right\},$$ (12)

$$W_{2}=\frac{c}{2}\left(I_{1}-3\right)+\sum_{i=1}^{4}\frac{c_{1i}}{4c_{2i}}\left\{e^{\left[c_{2i}{\left(I_{4i}-1\right)}^{2}\right]}-1\right\},$$ (13)

where ${\bar{I}}_{1}$ is the first isochoric invariant, ${\bar{E}}_{\alpha }$ is the effective fiber strain measure for family α , ${\bar{I}}_{4\left(\alpha \alpha \right)}$ is the isochoric fiber invariant for the family oriented along α , defined as ${\bar{I}}_{4\left(\alpha \alpha \right)}=a\left(\alpha \right)\cdot \bar{C}\cdot a\left(\alpha \right)$ , where $a\left(\alpha \right)$ is the unit fiber direction in the reference state, $k_{1}$ and $k_{2}$ are the positive material parameters of the anisotropic part, κ is a dispersion parameter of fiber orientations with 0 ≤ κ ≤ 1/3, $C_{01}$ and $C_{10}$ are the positive parameters of the isochoric isotropic part, and J is the relative volume change.

A curvilinear coordinate system is defined by solving the Laplace equation to determine the fiber orientation directions (Equations 14, 15):

$$\nabla \cdot \left(\nabla U\right)=0,$$ (14)

$$e=\frac{-\nabla U}{\left|-\nabla U\right|},$$ (15)

where U is the field function and e is the normalized vector field defining $a_{i}\left(\alpha \right)$ :

$$a_{1}=\frac{e}{\left|e\right|},$$ (16)

$$a_{2}=\frac{z-\left(z\cdot a_{1}\right)a_{1}}{\left|z-\left(z\cdot a_{1}\right)a_{1}\right|},$$ (17)

$$a_{3}=a_{1}\times a_{2}.$$ (18)

In Equations 16–18, *z* is a prescribed unit vector of the global axial direction.

The no-slip boundary condition is defined in Equation 19:

$$u_{f}\cdot n={u_{f}}_{tr}\cdot n,$$ (19)

where ${u_{f}}_{tr}$ is the translational velocity. The FSI interface states that the fluid displacements and the solid domain must be compatible. Tractions at this boundary must be at equilibrium, and the fluid must obey the no-slip condition, as shown in Equation 20:

$$u_{s}=u_{f},\upsilon_{s}=\upsilon_{f},\sigma_{s}\cdot {\hat{n}}_{s}=\sigma_{f}\cdot {\hat{n}}_{f}.$$ (20)

The FSI problem was solved using the ALE–FSI approach in the COMSOL Multiphysics software package (COMSOL Inc., Stockholm, Sweden). In the ALE method, the motion of the solid domain is described using a reference coordinate system that can be arbitrarily moved independently of both structural deformation and fluid motion. As a result, the fluid and solid domains can be consistently coupled while preserving the moving fluid–structure interface. We used the Navier–Stokes equations to describe the fluid motion. Then, we determined the total force acting on the solid (Equation 21):

$$f=n\cdot \left\{-pI+\left(\mu \left(\nabla u_{f}+{\left(\nabla u_{f}\right)}^{T}\right)\right)\right\},$$ (21)

where p is the pressure, μ is the dynamic viscosity, and n is normal to the solid body boundary.

The Navier–Stokes equations are solved in the spatial (deformed) frame, whereas the equations of solid mechanics are defined in the material (undeformed) frame, so the force must be transformed (Equation 22):

$$F=f\cdot \frac{d\upsilon }{dV},$$ (22)

where dυ and dV are the mesh element scale factors for the spatial frame and the material frame, respectively.

#### 2.4.1 Hemodynamic indices and post-processing

The same post-processing procedure was applied to both leaflet material models. The maximum velocity was evaluated in the ascending aorta, while pressure gradients were calculated between the inlet, the valve region, and the selected cross-section of the ascending aorta. The GOA was defined as the instantaneous open lumen area not occupied by the leaflets, whereas the EOA was estimated from the relationship between the inlet flow conditions and the maximum jet velocity downstream of the valve.

The geometric orifice area and effective orifice area were used to quantify the anatomical opening of the valve and were calculated using Equations 23, 24, respectively:

$$GOA\left(t\right)=A_{open}\left(t\right),$$ (23)

$$EOA=\frac{S_{inlet}\int_{t_{1}}^{t_{2}}u_{\max}dt}{\int_{t_{1}}^{t_{2}}u_{inlet}dt},$$ (24)

where $A_{open}\left(t\right)$ is the instantaneous open lumen area not occupied by the leaflets, $S_{inlet}$ is the area of the aortic root inlet, $u_{\max/inlet}$ is the corresponding maximum and inlet velocities.

Vortical flow structures were assessed using the vorticity magnitude and the Q-criterion. Vorticity was used to characterize the local rotation of the flow, while the Q-criterion was used to identify three-dimensional vortex cores in the aortic root and ascending aorta.

The vorticity vector was calculated using Equation 25:

ω=∇×u, (25)

where u is the fluid velocity vector. The Q-criterion was used to identify vortex-dominated regions and was defined in Equation 26:

$$Q=\frac{1}{2}\left({\left\|Ω\right\|}^{2}-{\left\|S\right\|}^{2}\right),$$ (26)

where S and Ω are the symmetric strain-rate tensor (Equation 27) and the antisymmetric rotation-rate tensor (Equation 28), respectively:

$$S=\frac{1}{2}\left(\nabla u+\nabla u^{T}\right),$$ (27)

$$Ω=\frac{1}{2}\left(\nabla u-\nabla u^{T}\right).$$ (28)

WSS was computed from the viscous stress tensor as the tangential component of the traction acting on the leaflet surface. These quantities were extracted at representative time instants of the cardiac cycle and were used to compare the influence of the Holzapfel (2005) model with that of the four-fiber-family material model on valve hemodynamics.

Wall shear stress is defined from the viscous stress tensor, as shown in Equations 29, 30:

$$T=\kappa \cdot \hat{n}$$ (29)

$$WSS=\left|T-\left(T\cdot \hat{n}\right)\hat{n}\right|,$$ (30)

where $\hat{n}$ is the wall normal direction. WSS represents the tangential component of the force exerted by the blood flow on the cardiovascular structures.

#### 2.4.2 Mesh convergence and numerical solver settings

Leaflet coaptation was modeled in COMSOL Multiphysics using a penalty-based contact formulation. In this approach, the contact constraint is enforced approximately by adding a distributed compressive stiffness that becomes active only under compression, which is numerically robust for transient multiphysics simulations but allows a small amount of interpenetration. In the present ALE–FSI setting, complete geometrical closure of the leaflet gap can lead to severe distortion or collapse of the fluid mesh in the inter-leaflet region. Therefore, a minimal normal clearance was prescribed between the contacting leaflet surfaces, set equal to the minimum element size in the fluid domain. This clearance enables transfer of contact forces without fully collapsing the fluid layer, thereby maintaining mesh integrity and solver stability during the valve closure.

Computational meshes for both the fluid and solid domains were generated, and a mesh-convergence study was conducted. The mesh sensitivity analysis was performed by comparing several successively refined meshes under identical boundary conditions, material parameters, and solver settings. The convergence assessment was based on global mechanical and hemodynamic quantities, including peak flow velocity, maximum leaflet deformation, pressure gradient, WSS, and von Mises stress. The results of the mesh sensitivity study are summarized in Table 1. The coarser meshes showed noticeable deviations from the finest mesh, especially for maximum velocity and WSS, where the differences reached 9.41% and 13.04%, respectively. However, when the mesh was refined from the penultimate to the finest variant, the normalized values of maximum velocity, maximum WSS, and maximum von Mises stress reached 99.41%, 99.13%, and 99.81% of the finest-mesh solution, corresponding to relative differences of only 0.59%, 0.87%, and 0.19%, respectively. These results indicate that further mesh refinement produced negligible changes in the main mechanical and hemodynamic quantities, confirming mesh-independent solutions. The final mesh consisted of 634,582 elements in the fluid domain and 248,442 elements in the solid domain, yielding a total of 883,024 elements (Figure 3B). The mesh included tetrahedral, prismatic, triangular, quadrilateral, edge, and vertex elements. In the fluid domain, five boundary-layer (inflation) layers were used, with a growth factor of 1.2. The average element quality was 0.68, and the minimum element quality was 0.12.

**TABLE 1:** Mesh sensitivity analysis based on maximum velocity, WSS, and von Mises stress.

Mesh case	Mesh elements	Maximum velocity, m/s	Relative difference, %	Maximum WSS, Pa	Relative difference, %	Maximum von Mises stress, kPa	Relative difference, %
Mesh 1	137,752	1.55	9.41	50	13.04	510	3.04
Mesh 2	268,728	1.76	2.94	60	4.31	520	1.14
Mesh 3	494,388	1.7	0.59	57	0.87	525	0.19
Mesh 4	883,024	1.71	0	57.5	0	526	0

Within the ALE–FSI formulation, a mesh updating algorithm was used. Mesh motion in the fluid domain was described using the ALE approach, and automatic remeshing was enabled in COMSOL Multiphysics to prevent excessive element distortion. The element distortion was monitored using the isochoric first invariant of the mesh deformation tensor, as shown in Equation 31:

$$I_{1,\text{iso}}=J_{m}^{-2/3}\text{tr}\left(F_{m}^{T}F_{m}\right),$$ (31)

where $F_{m}=\partial x/\partial X_{m}$ is the mesh deformation gradient, $J_{m}=\det\left(F_{m}\right)$ is the corresponding Jacobian, $X_{m}$ denotes the mesh coordinates in the reference configuration, and x denotes the current spatial coordinates. Remeshing was performed when the distortion criterion satisfied Equation 32:

$$D=\sqrt{I_{1,\text{iso}}^{\max}}>2,$$ (32)

where $I_{1,\text{iso}}^{\max}$ is the maximum value over all mesh elements. After remeshing, the solution was mapped onto the updated mesh. This procedure preserved acceptable element quality near the moving leaflets and improved the stability of the coupled simulation during valve opening and closure.

For the structural domain, solid mechanics was discretized with linear interpolation of the displacement field. For the fluid domain, a stabilized P_2_+P_1_ finite-element pair was employed, i.e., continuous piecewise-linear shape functions for both velocity and pressure. The problem was solved using an implicit coupled scheme with sub-iterations at each time step. The fluid and solid subproblems were solved sequentially until interface consistency was achieved; relaxation was enabled at the beginning of the cardiac cycle and during phases with rapid deformation to improve robustness. At each time step, sub-iterations were performed until a relative residual tolerance of $10^{-4}$ was reached for fluid velocity and pressure and $10^{-4}$ for solid displacements, with a maximum of 20 sub-iterations per time step. Time integration used a second-order implicit backward differentiation formula (BDF) for the fluid and an implicit Newmark-type scheme for solid mechanics. The time step was chosen adaptively in the range of $10^{-6}$ to $10^{-3}$ s.

## 3 Results

### 3.1 Biaxial tensile data analysis

**FIGURE 4:**
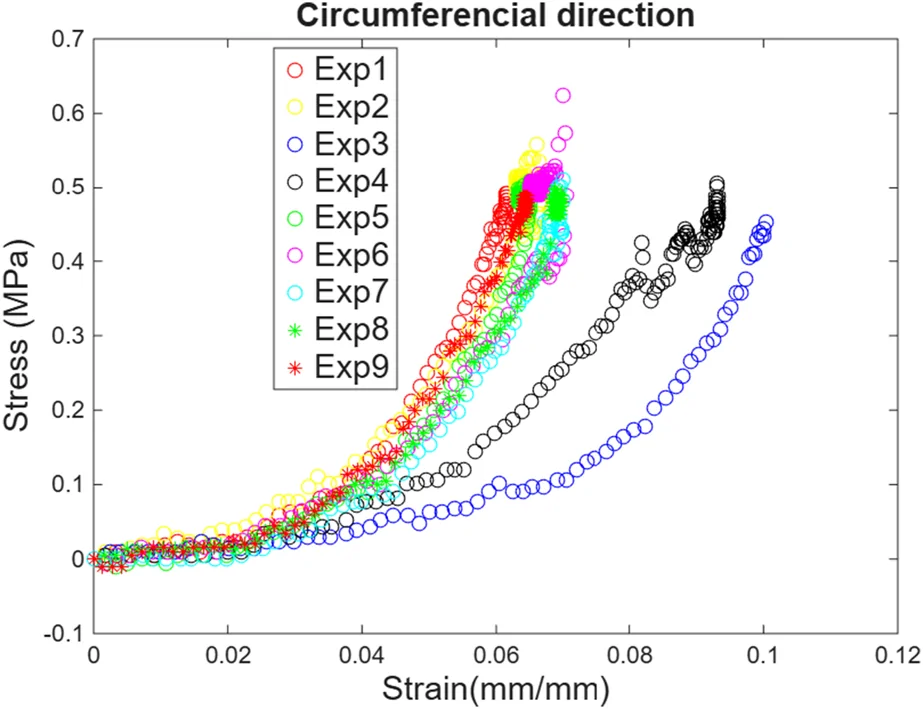
Experimental results in the circumferential direction.

**FIGURE 5:**
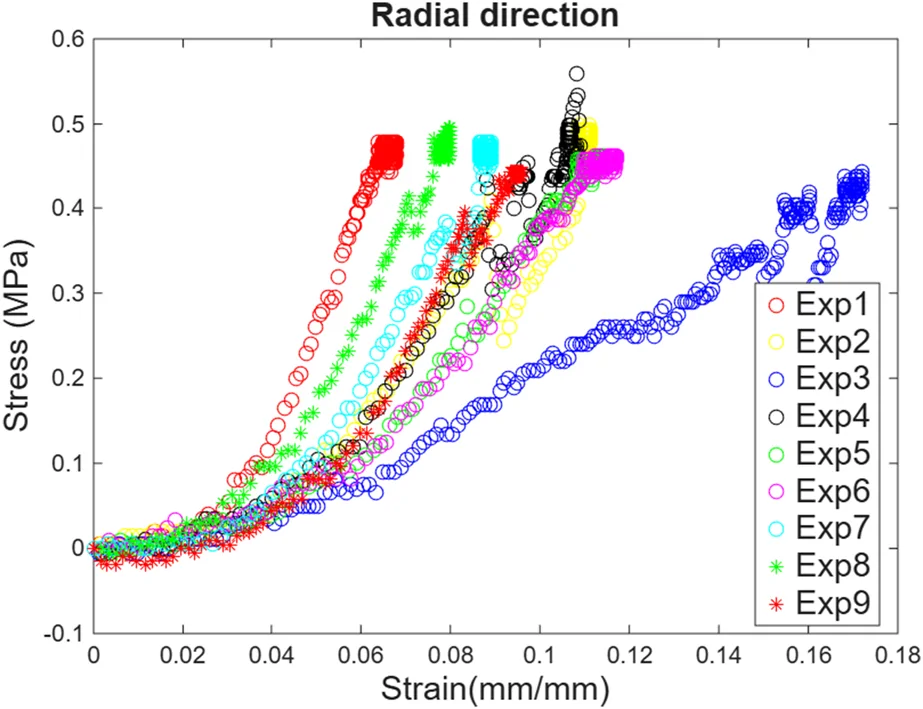
Experimental results in the radial direction.

Engineering stress and strain were calculated separately for each loading direction to present the directly measured biaxial response. For constitutive parameter identification, the raw force–displacement data were converted to finite deformation measures. The deformation gradient F was determined from the measured displacements, from which the Green–Lagrange strain tensor, ( $E=\text{1/2}\left(F^{T}F-I\right)$ ), and the second Piola–Kirchhoff stress tensor, ( $S=JF^{\left(-1\right)}\sigma F^{\left(-T\right)}$ ), were calculated, where J=det(F) and **σ** is the Cauchy stress tensor. These finite-deformation measures were used to identify the parameters of all ten hyperelastic constitutive models. The engineering stress–strain curves presented in Figures 4, 5 are used to visualize the experimental tissue response and to compare it with the constitutive model predictions, whereas the quantitative material parameters listed in Supplementary Material 3 and subsequently used in the FSI simulations were derived from finite deformation measures.

Through virtual inspection in Supplementary Table S1 (see Supplementary Material S3), it is evident that the circumferential strain at maximum applied stress is lower than that in the radial direction (Figures 12, 13). This observation suggests that the radial direction contains fewer fibers resisting deformation than the circumferential direction. A similar response was observed for the pericardium in the previous findings (Sulejmani et al., 2019).

The average strain obtained in the circumferential direction was 0.0696 ± 0.0102 mm, and that in the radial direction was 0.1060 ± 0.0297 mm, which is significant (p = 0.0033). These results show that the circumferential direction resists deformation better than the radial direction at a maximum applied stress of 0.48 MPa.

At 50% of maximum applied stress, the pericardium shows coupling behavior with the circumferential strain of 0.0587 ± 0.0122 and the radial strain of 0.0585 ± 0.0073, which is insignificant (p = 0.9609). At approximately 0.24 MPa, the pericardium deforms uniformly in both directions, with the circumferential direction slightly resisting the deformation better than the radial direction. The selection of nine samples was based on an effect size of 1.6393, calculated as the ratio of the difference in means to the pooled estimated standard deviation between the circumferential and radial directions. A significance level of 0.05 and a required power of 0.9 resulted in a sample size of 9. Parameters of constitutive relations and statistical metrics are presented in Supplementary Material S3.

**FIGURE 6:**
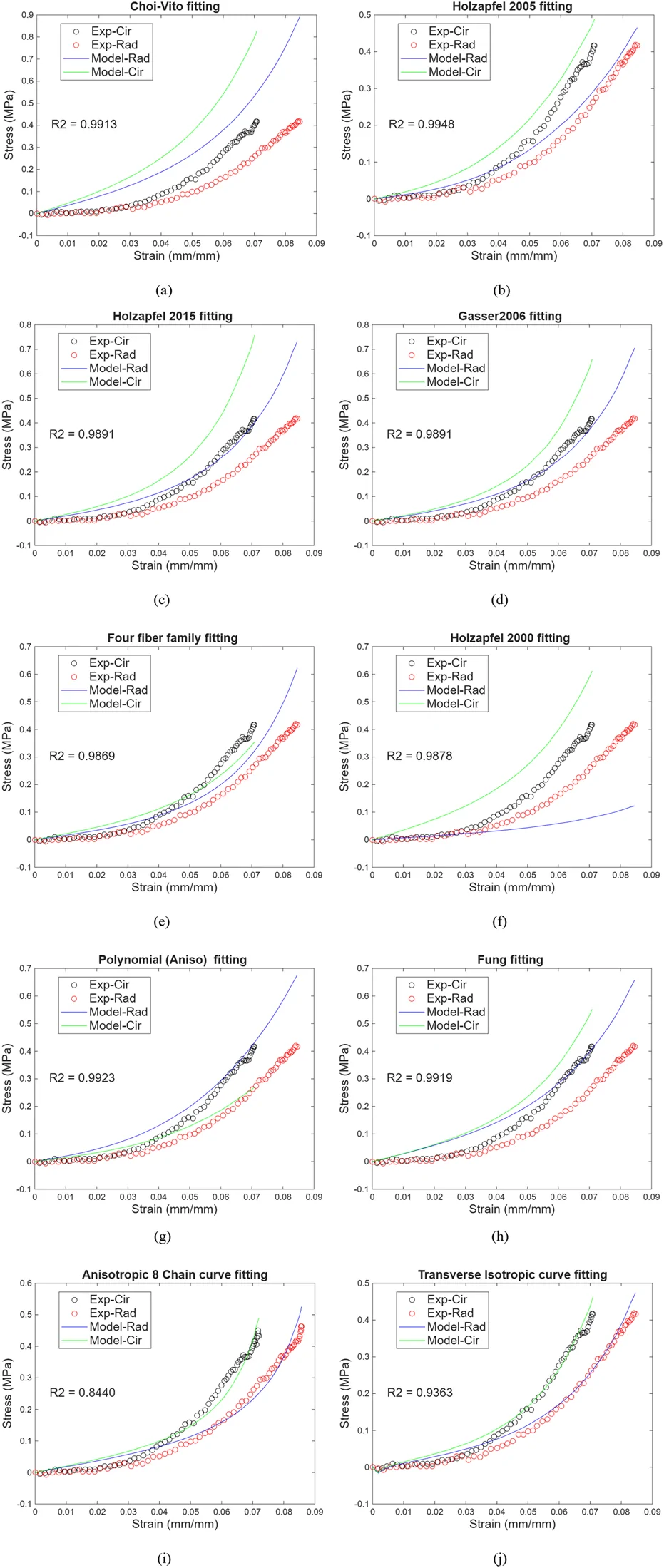
Constitutive models fitted to experimental data: **(A)** Choi–Vito; **(B)** Holzapfel 2005; **(C)** Holzapfel 2015; **(D)** Gasser 2006; **(E)** Four-fiber family; **(F)** Holzapfel 2000; **(G)** Polynomial anisotropic; **(H)** Fung; **(I)** Anisotropic 8-chain; and **(J)** Transversely isotropic.

To evaluate the performance of constitutive models, an evaluation index was employed by using the average coefficients of determination of all models (Figure 6).

**FIGURE 7:**
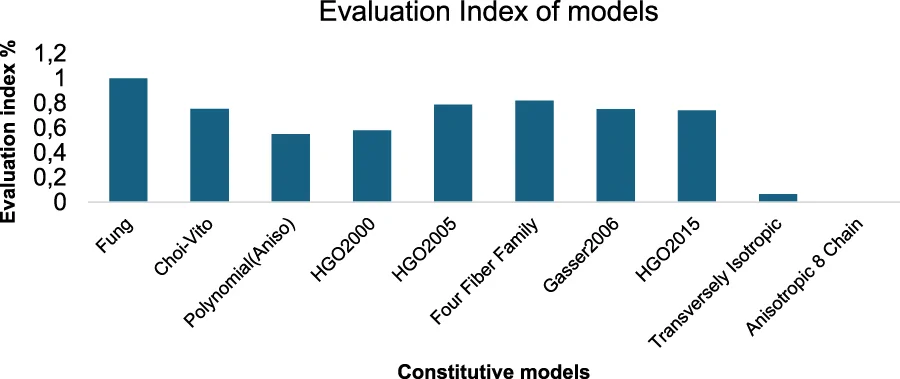
Evaluation index of the fitted model to experimental data using the averaged coefficients of determination.

Figure 6 shows a plot of the average experimental results fitted with 10 hyperelastic anisotropic constitutive models, and the average material parameters from all 10 constitutive models were used to fit on averaged independent experimental data. Through virtual inspection of Figure 6a, the Choi–Vito model does not adequately capture the circumferential and radial strains, unlike the Holzapfel and Ogden (2015) model shown in Figure 6c. The model does not fit well in both directions. The Gasser (2006) model in Figure 6d shows good results in capturing circumferential deformation but lacks precision in the radial direction. Holzapfel (2000), Fung, and the polynomial models shown in Figures 6f–h do not perform well in predicting material response in both directions. Both the Holzapfel (2005) model and the four-fiber-family model shown in Figures 7b,e show a superior response in both directions.

**FIGURE 8:**
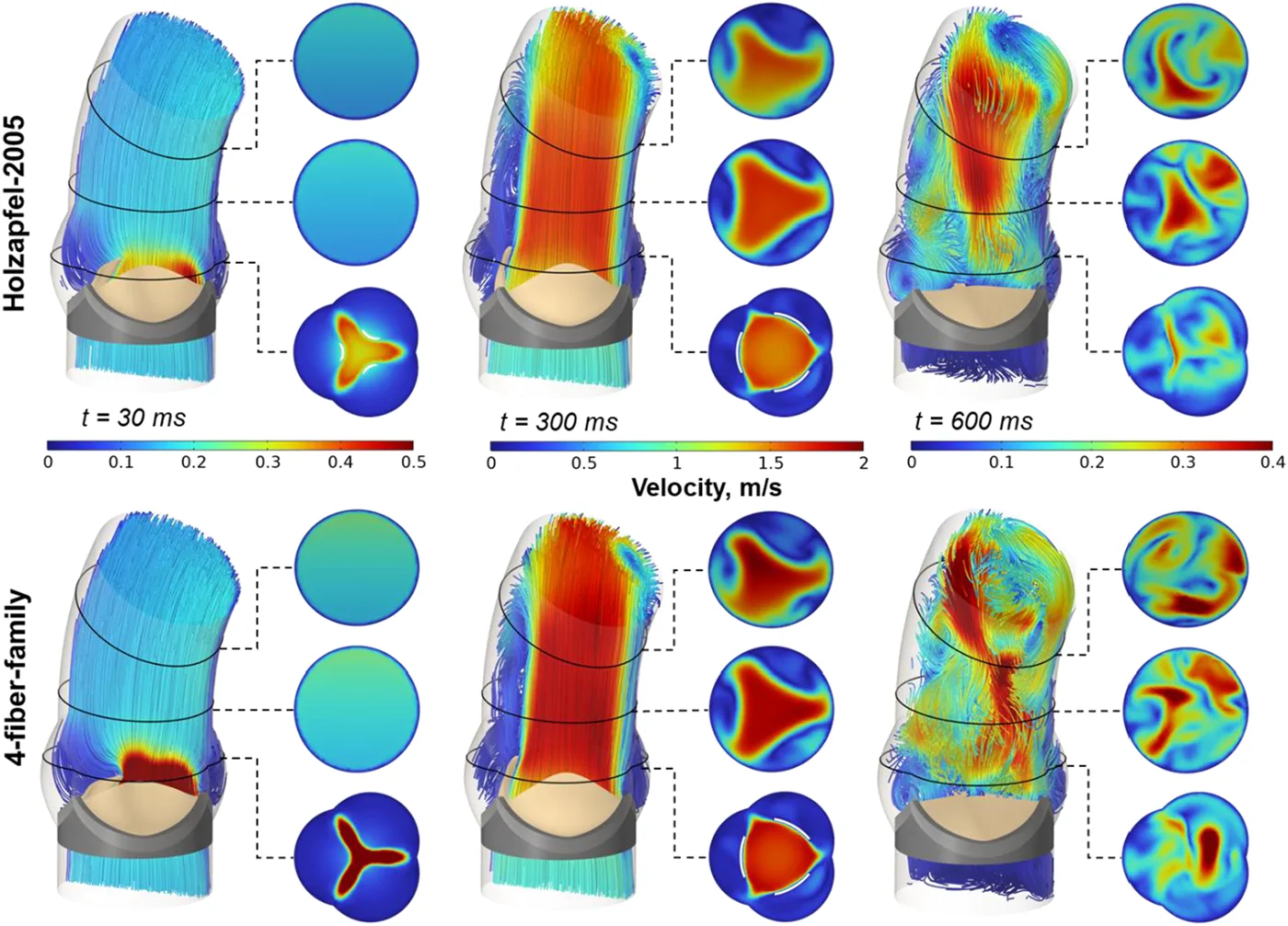
Streamlines and velocity magnitude distributions for the Holzapfel (2005) and four-fiber-family leaflet material models.

Figure 8 shows an evaluation index using the coefficient of determination. The evaluation index results for selected models are as follows: Fung’s model 100%, polynomial model 55%, Choi–Vito’s model 75%, Gasser (2006) model 75%, Holzapfel (2000) model 58%, Holzapfel (2015) model 74%, Holzapfel (2005) model 79%, four-fiber-family model 82%, transversely isotropic model 6.5%, and anisotropic 8-chain model 0%.

### 3.2 FSI aortic valve hemodynamics

As part of the numerical experiments, two material models, the Holzapfel (2005) model and the four-fiber-family model, were examined to describe the hemodynamics of the aortic valve. Although the Fung model achieved the highest evaluation index based on the coefficient of determination for equibiaxial curve fitting, it was not selected for subsequent FSI simulations. The primary reason is that the Fung model is a phenomenological anisotropic formulation that does not explicitly represent the underlying collagen fiber architecture of the pericardium. In contrast, the Holzapfel (2005) and four-fiber-family models are structurally motivated fiber-reinforced models. Their explicit fiber definitions enable the mapping of fiber directions onto the curved leaflet geometry using a curvilinear coordinate system, independent control of the mechanical contributions of the matrix and fibers, which is essential for capturing the toe-to-heel transition under the complex, multiaxial loading experienced by a working valve, and prediction of fiber-level stresses relevant to durability and mechanobiology. Furthermore, these fiber-explicit models are widely used in the cardiovascular FSI literature, facilitating the comparison of our results with those of previous studies. Therefore, model selection for FSI was based not solely on curve-fitting metrics but on structural plausibility, ability to generalize beyond the equibiaxial loading protocol, and mechanistic interpretability. The evaluation index was used to down-select from 10 models to a smaller set of well-fitting candidates; the final choice for FSI was then driven by the specific requirements of fiber-reinforced computational modeling.

The model parameters were derived from biaxial tensile tests, in which these formulations were found to be the most suitable for capturing the hyperelastic anisotropic behavior of pericardial tissue. The influence of these models on various mechanical and hemodynamic characteristics of the aortic valve was assessed, including flow velocity, leaflet extension, displacement, von Mises equivalent stress, volumetric strain, and wall shear stress.

Figure 8 presents streamlines and the velocity magnitude in the aortic root and ascending aorta for the two leaflet material models. The top row corresponds to the Holzapfel (2005) model, and the bottom row to the four-fiber family model. Three representative instants of the cardiac cycle are shown: leaflet motion onset at 30 ms, peak systole at 300 ms, and leaflet closure at 600 ms. The velocity magnitude is color-coded, and a separate velocity scale is used at each time instant. Cross sectional velocity magnitude maps are reported in the xOy plane at heights of 46 mm, 35 mm, and 18 mm from the aortic root base, from top to bottom. At 30 ms, acceleration is confined to the immediate post-valve region, while the distal sections remain weak and nearly axisymmetric. At 300 ms, a pronounced systolic jet develops and becomes eccentric due to the aortic curvature, with secondary flow features evident from nonuniform and three lobed patterns just downstream of the valve and from the progressive redistribution of the high-velocity core along the ascending aorta.

The overall flow topology is similar for both material models at 30 ms and 300 ms, whereas the four-fiber-family model yields slightly higher velocities and a more extended high velocity core than the Holzapfel (2005) model. At 600 ms, the jet breaks down into multiple vortical structures and recirculation zones during closure, while the velocity magnitude remains below 0.4 m/s, indicating that decaying rotational motion dominates the late phase of the cycle.

**FIGURE 9:**
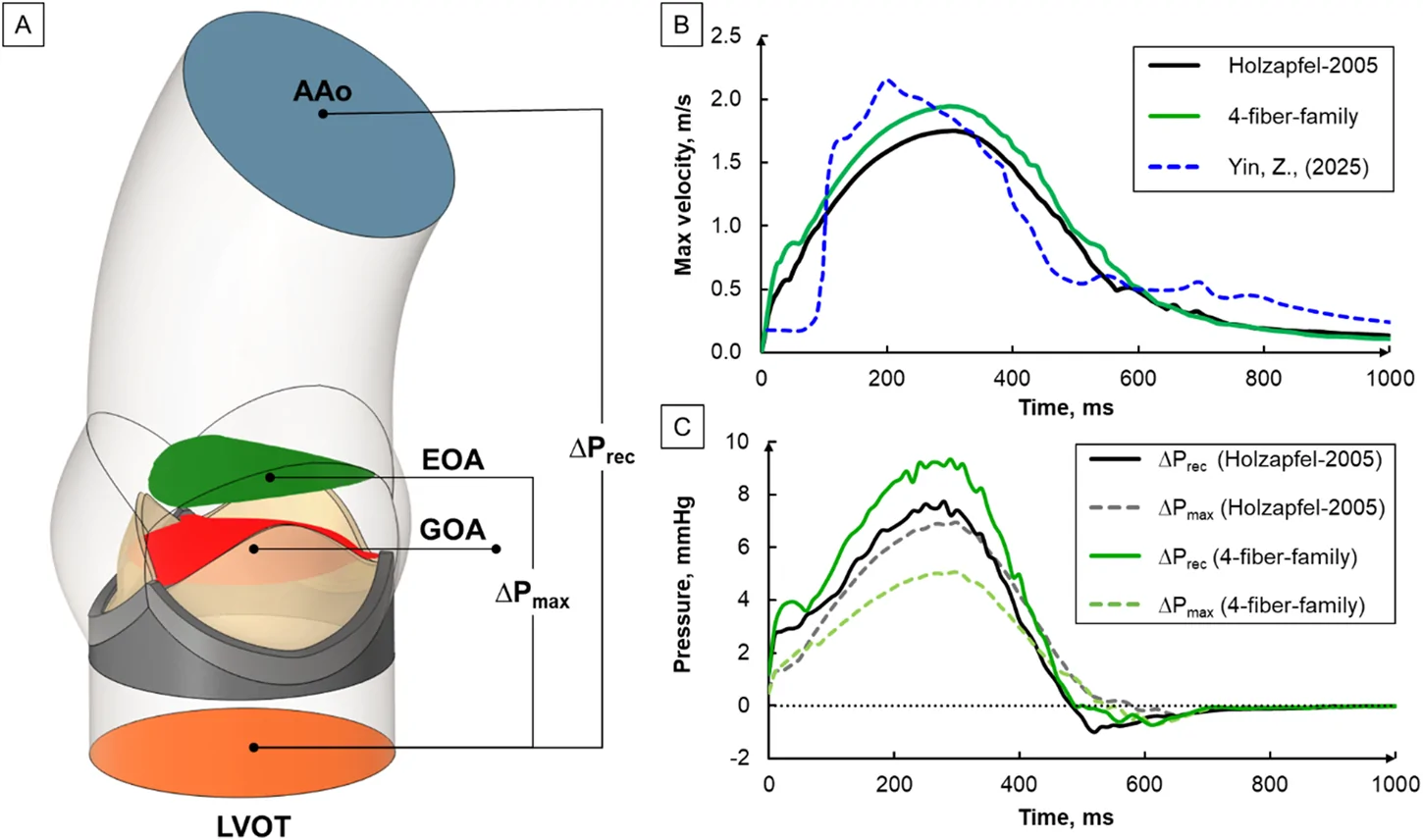
**(A)** Scheme of the aortic root with key surfaces for determining hemodynamics indices; **(B)** maximum velocity comparing with that of the *in silico* model by Yin et al. (2024); **(C)** maximum and recovery pressure gradients for both models.

Figure 9A shows the aortic root schematic with the key planes and surfaces used to compute hemodynamic indices. The LVOT inlet plane, the planes used to determine GOA and EOA, and the AAo cross-section in the ascending aorta are indicated. These locations were used to evaluate the maximum pressure gradient ΔP_max_ and the pressure recovery gradient ΔP_rec_. Figure 9B reports the time evolution of the maximum velocity in the ascending aorta for the Holzapfel (2005) and four-fiber-family leaflet models. The peak velocity was 1.77 m/s at 323 ms for the Holzapfel (2005) model and 1.94 m/s at 315 ms for the four-fiber-family model. The difference was 0.17 m/s, corresponding to 9%, while the waveform shapes remain qualitatively consistent across models, with a rapid systolic increase followed by a gradual decay during ejection. The magnitude is comparable to the results reported by Yin et al. (2024), where the maximum reaches 2.15 m/s at 201 ms, and the earlier peak may reflect differences in boundary conditions and loading profiles. Figure 9C presents ΔP_max_ and ΔP_rec_ for both models. The peak pressure gradient reaches 6.8 mmHg for the Holzapfel (2005) model and 5.1 mmHg for the four-fiber-family model. The pressure recovery gradient ranged from 0 mmHg to 7.75 mmHg for the Holzapfel (2005) model and from 0 mmHg to 9.34 mmHg for the four-fiber-family model. The obtained values fall within the range expected for normal aortic valve function.

The difference between the two pressure gradients can be attributed to model-dependent leaflet kinematics and jet formation. In the four-fiber family case, the smoother orifice shape and more pronounced high-velocity jet core promote stronger downstream pressure recovery after the jet contraction region. Therefore, despite a lower maximum pressure gradient across the valve, this model shows a higher-pressure recovery gradient. In the Holzapfel (2005) model, the flow is more localized and dissipative, which reduces downstream pressure recovery and results in more similar values of ΔP_max_ and ΔP_rec_.

**FIGURE 10:**
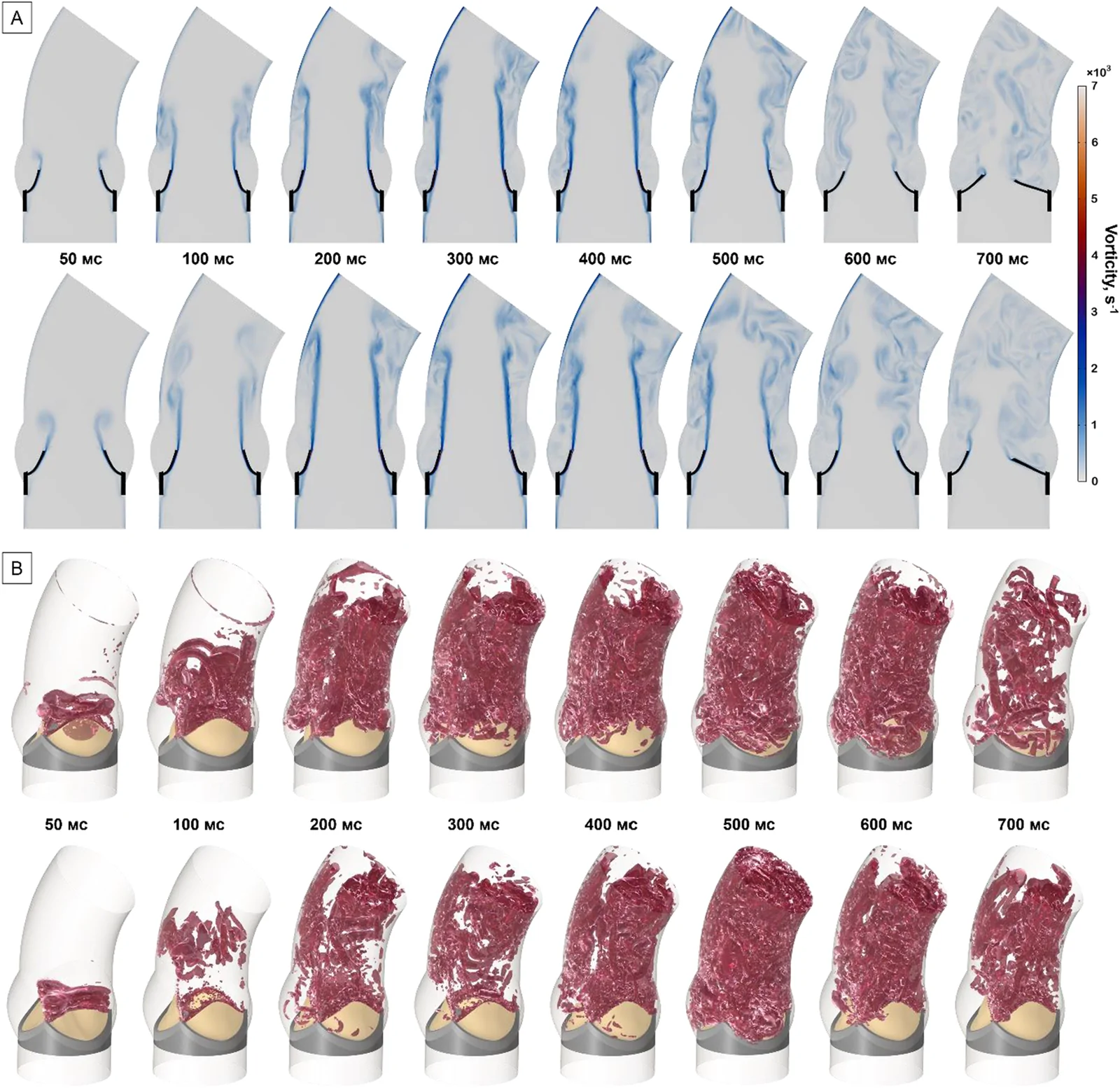
Comparison of flow structures in the aortic root at representative instants of the cardiac cycle for the Holzapfel (2005) and four-fiber family leaflet material models: **(A)** vorticity magnitude distributions. **(B)** Vortical structures visualized using the Q-criterion; the upper row corresponds to the Holzapfel (2005) model, and the lower row corresponds to the four-fiber-family model.

Figure 10A shows the vorticity magnitude in a longitudinal plane within the aortic root and ascending aorta at representative instants of the cardiac cycle. In early systole, vorticity peaks concentrate near the leaflet free edges and within the sinuses, indicating the onset of shear-layer formation and jet development. Around peak systole, the high-vorticity region elongates along the eccentric jet, whereas during leaflet closure, the field becomes more fragmented, consistent with jet breakdown and recirculation Figure 10B. shows the corresponding Q-criterion isosurfaces, which reveal compact vortex cores near the leaflets and a more complex vortical organization during closure.

**FIGURE 11:**
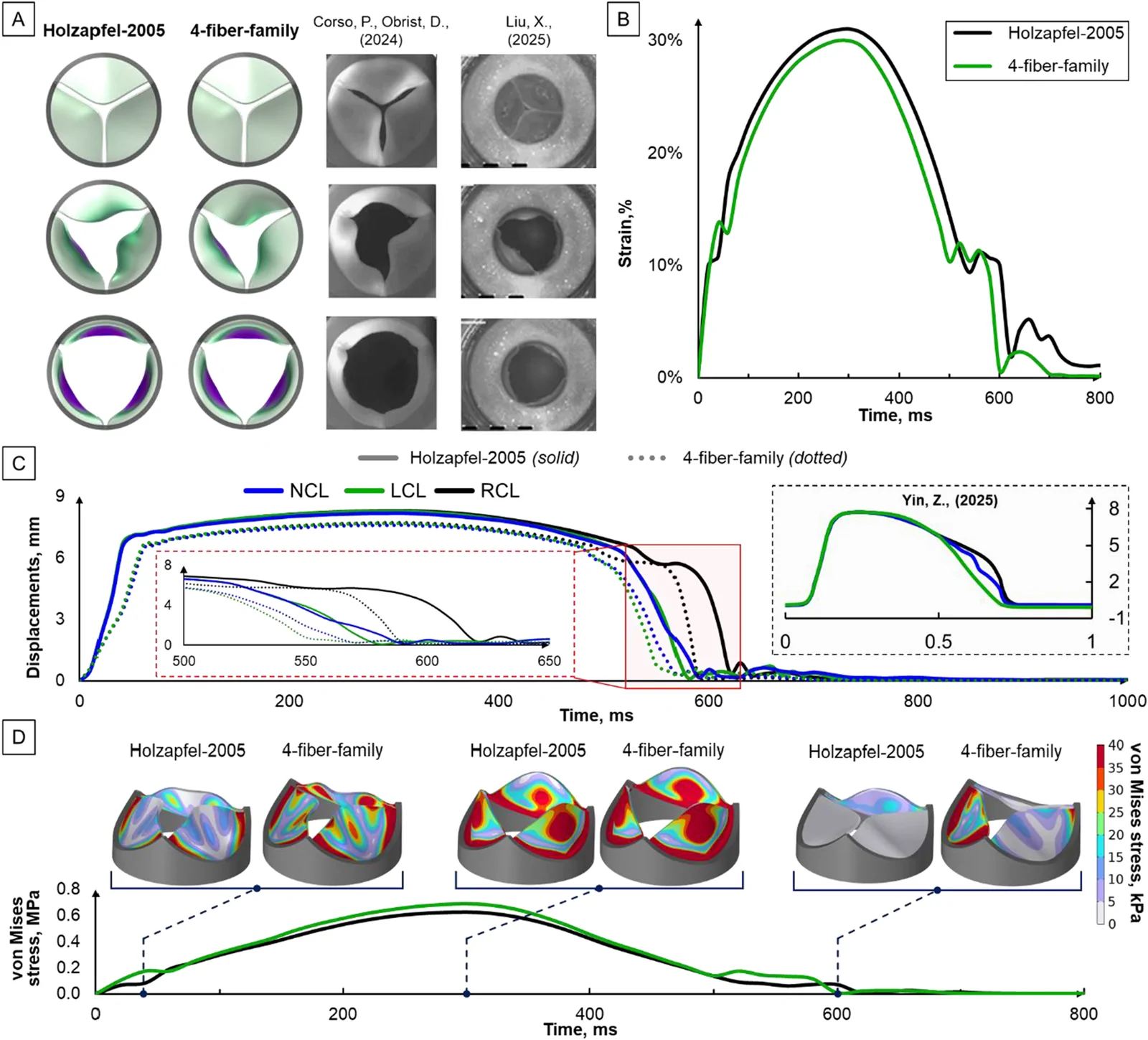
Leaflet kinematics and commissural displacements. **(A)** Verification with experimental data (Corso and Obrist, 2024; Liu et al., 2025); **(B)** maximum strain during the cardiac cycle; **(C)** maximum displacements for each leaflet: NCL, non-coronary leaflet, LCL, left coronary leaflet, RCL, right coronary leaflet, and verification within the in silico model (Yin et al., 2024); **(D)** maximum von Mises stress and distributions of stresses on valves for both models.

Figure 11 provides a combined comparison of aortic valve leaflet dynamics obtained from the two constitutive material models. Panel A shows representative valve configurations in the closed state, during opening, and at maximum opening for the Holzapfel (2005) and four-fiber-family models, together with experimental images from Corso and Obrist (2024) and Liu et al. (2025) used for qualitative verification of the orifice shape and the coaptation pattern. Both models reproduce a tri-radiate closure line in diastole and a three-lobed orifice in systole, while the four-fiber family model visually exhibits a slightly larger opening and a smoother lumen shape at peak opening.

Figure 11B reports the time evolution of the peak leaflet strain over the cycle. In both models, strain rapidly increases in early systole, reaches a maximum of approximately 30% during ejection, and decreases during closure. Differences become apparent near end-systole: the Holzapfel (2005) model maintains a higher strain level on the plateau and shows a more prolonged decay after closure, whereas the four-fiber-family model displays a faster decrease with smaller residual fluctuations.

Figure 11C shows commissural displacements for the three leaflets: non-coronary leaflet (NCL), left coronary leaflet (LCL), and right coronary leaflet (RCL). Solid lines correspond to the Holzapfel (2005) model, and dotted lines to the four-fiber family model. The curves share the same structure, with a rapid increase in early systole, a plateau during ejection, and a steep decrease during closure. The zoomed inset (500–650 ms) highlights the closure phase and small post-contact oscillations, which are more pronounced for the Holzapfel (2005) model and may reflect differences in stiffness and contact-driven dynamics. The right inset compares the trends with Yin et al. (2024), confirming a similar order of magnitude and waveform, with potential phase differences attributable to boundary conditions and geometry.

Figure 11D presents the time evolution of the von Mises equivalent stress, together with stress maps at selected instants. Peak stresses occur during active opening and the ejection plateau, consistent with the largest membrane tension at maximum opening. Elevated stresses concentrate near the commissures and along the annular attachment line, extending toward the free edge. Overall, the stress time profiles are similar across models, while the four-fiber family model yields slightly higher systolic stresses and a sharper stress decay as the valve transitions to closure.

**FIGURE 12:**
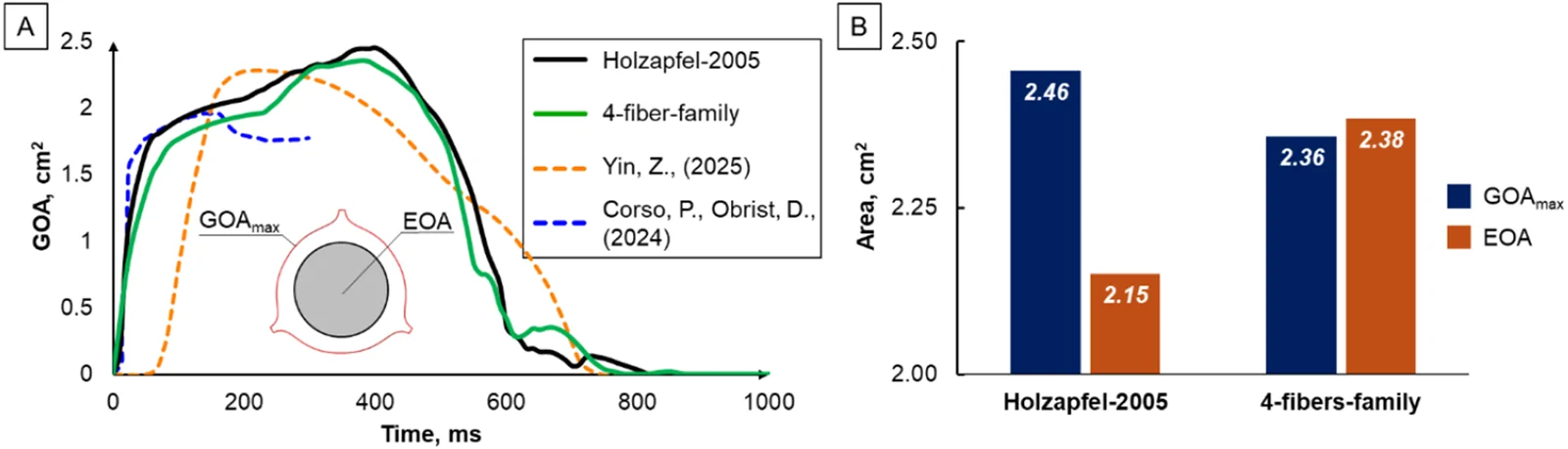
Comparison of flow structures in the aortic root at representative instants of the cardiac cycle for the Holzapfel 2005 and Four-fiber family leaflet material models: **(a)** vorticity magnitude distributions. **(b)** vortical structures visualized using the Q-criterion, the upper row corresponds to the Holzapfel 2005 model, and the lower row corresponds to the Four-fiber family model.

Figure 12A shows the time evolution of GOA for the Holzapfel (2005) and four-fiber family models. Both models exhibit a rapid increase during early systole, a plateau near peak opening, and a subsequent decrease during closure. The peak GOA equals 2.46 cm^2^ for the Holzapfel (2005) model and 2.36 cm^2^ for the four-fiber family model (Figure 12B). The EOA values were 2.15 cm^2^ for the Holzapfel (2005) model and 2.38 cm^2^ for the four-fiber family model. For the four-fiber family model, the estimated EOA was very close to the GOA, with a difference of less than 1%; therefore, these values should be interpreted as practically comparable rather than as a physical excess of EOA over GOA.

**FIGURE 13:**
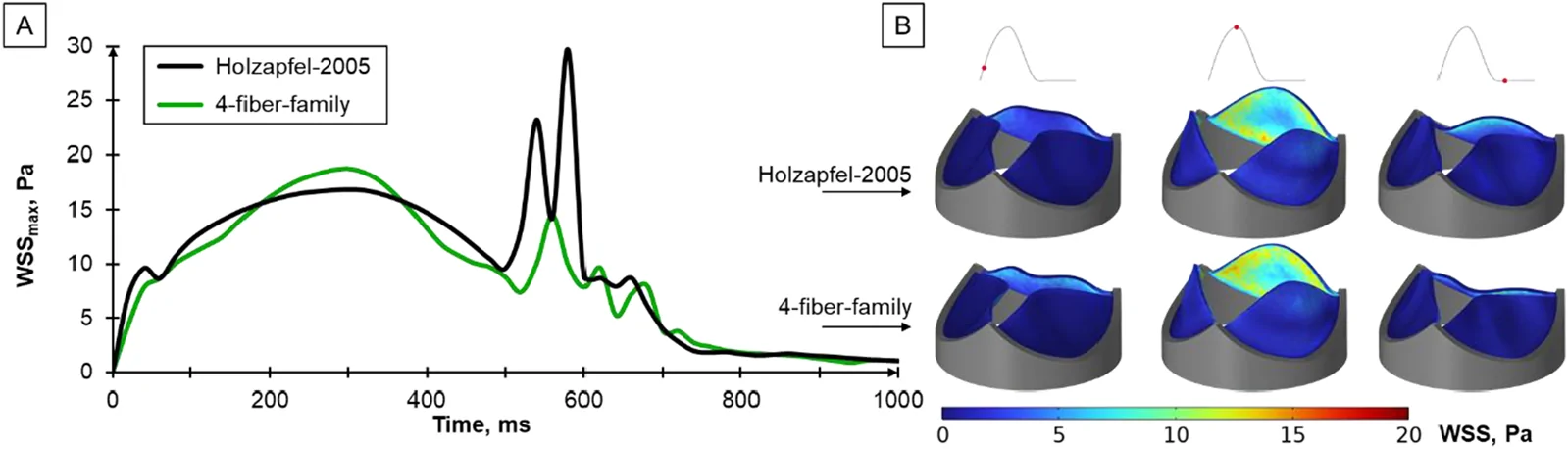
Peak wall shear stress **(A)** and leaflet WSS maps **(B)** for the Holzapfel (2005) and four-fiber family models.

Figure 13 compares the shear stresses of the leaflet surface for the two material models. Panel A shows the time evolution of the peak wall shear stress, $WSS_{\max}$ , evaluated over the leaflet surfaces throughout the cardiac cycle. A rapid increase is observed in early systole, followed by a plateau during ejection that corresponds to a relatively steady jet regime. Toward end-systole and during closure, pronounced peaks in $WSS_{\max}$ emerge, reflecting locally increased near-wall velocity gradients as the orifice area decreases and the flow reorganizes near the leaflets. These closure-phase peaks are more pronounced for the Holzapfel (2005) model, reaching approximately 25–30 Pa at 600 ms, whereas the four-fiber family model yields lower and smoother peak values. During diastole, $WSS_{\max}$ rapidly decreases to small values, consistent with the attenuation of motion after valve closure.

Figure 13B presents spatial WSS maps on the leaflet surfaces at the selected instants indicated above the renderings. For both models, the highest WSS regions are primarily located near the free edge and around the commissures, where the strongest tangential velocity gradients develop, while the central leaflet regions exhibit lower values. During systole, elevated WSS bands extend along the main flow direction, whereas during the transition to closure, the distributions become more heterogeneous with localized maxima. Differences between the two models appear in the degree of high-WSS concentration and spatial extent, consistent with the previously observed differences in leaflet kinematics and closure dynamics.

These findings are consistent with those of Tsolaki et al. (2023), showing that mineralization and collagen network alterations in failed bioprosthetic valves spatially coincide with the regions of elevated hemodynamic and biomechanical indicators, including TAWSS, OSI, RRT, and TSVI. Therefore, the WSS-based analysis in the present study complements GOA, EOA, and pressure gradient assessment by identifying leaflet regions exposed to potentially unfavorable shear conditions, especially near the free edges and commissures during valve closure.

Clinical interpretation of computed hemodynamic metrics: The peak ascending-aorta velocity obtained in this study, 1.77 m/s for the Holzapfel (2005) model and 1.94 m/s for the four-fiber-family model, lies within the range typically reported for normally functioning aortic valves and is markedly below the values associated with clinically significant stenosis. Consistently, the peak pressure gradients remain low, with ΔP_max_ of 6.8 mmHg and 5.1 mmHg for the Holzapfel (2005) and four-fiber-family models, respectively. The effective orifice area values, 2.15–2.38 cm^2^, indicate an unobstructed opening under the prescribed physiological loading and remain well above thresholds commonly used to grade moderate-to-severe aortic stenosis. Although GOA reflects the anatomical opening, EOA additionally captures jet contraction and energy losses, which makes it more sensitive to leaflet deformation and flow–structure coupling.

## 4 Discussion

### 4.1 Experimental study

Equibiaxial tensile tests were performed to characterize the Landrace pig’s pericardium collected from a local abattoir. The results show that the Landrace pig pericardium is an anisotropic hyperelastic material. These findings are consistent with those of Caballero et al. (2017), although their results were for a chemically treated porcine pericardium. The results demonstrate reliability, with only a 10% probability of failing to detect a true effect. The distinction between circumferential and radial directional behaviors was established with 90% statistical power, further supporting the robustness of the data.

The relevance of this study arises from a lack of biaxial tensile test characterization of native porcine pericardium because most studies have performed uniaxial tests. At a stress of 0.5 MPa, the circumferential strain of chemically treated porcine pericardium was found to differ from our finding, with our value and their value being 7% and 5%, respectively; however, the radial direction response was approximately the same for both our study and the above-mentioned study, with both studies recording approximately 11% strain at a stress of 0.5 MPa.

The differences observed in the fitting performance of the constitutive models can be explained by the way each model represents the isotropic matrix response, the anisotropic fiber contribution, and the transition between these two mechanical regimes. Since porcine pericardium is a fiber-reinforced soft tissue, the ability of a model to capture both the initial compliant response and the later stiffening behavior under biaxial stretch is critical.

The Fung model was able to represent the nonlinear response of the tissue because of its exponential formulation. However, its stiffness matrix influences both the isotropic and anisotropic components of the material response. This coupling limits the model’s ability to independently describe the direction-dependent behavior of the pericardium. As a result, the model may fit the general nonlinear trend but may not fully capture the differences between the circumferential and radial responses.

The Choi–Vito model also uses an exponential strain-energy formulation. However, the ground stiffness matrix has a strong influence on both the isotropic and anisotropic parts of the response. This may reduce the model’s ability to accurately predict the anisotropic behavior of the tissue, particularly under biaxial loading where the fiber orientation and stretch direction strongly affect the mechanical response. Therefore, although the model can capture soft-tissue nonlinearity, its performance may be limited when the tissue shows strong directional dependence.

The Holzapfel (2000) model includes both isotropic and anisotropic components in the strain-energy density function. In this model, the anisotropic fiber contribution is activated through the fiber stretch invariant $I_{4i}$ . When $I_{4i}$ is less than or equal to 1, the anisotropic component is not fully activated, allowing the model to represent the initial toe region of the material response. However, once $I_{4i}$ becomes greater than 1, the anisotropic component is introduced more strongly. This sudden activation may limit the model’s ability to smoothly capture the transition from matrix-dominated behavior to fiber-dominated behavior during tensile deformation.

The Holzapfel (2005) model improved the representation of the experimental biaxial tensile response by introducing the weighting function ρ , which provides better coupling between the isotropic matrix and anisotropic fiber contributions. This modification allows the model to describe the gradual transition between the toe region and the fiber-dominated stiffening region more effectively. Consequently, the Holzapfel (2005) model provided a better representation of the mechanical behavior of the porcine pericardium under biaxial stretch, particularly where both matrix deformation and fiber recruitment contribute to the overall tissue response (Holzapfel et al., 2005).

The results show that the fitting performance of the constitutive models was not only dependent on their mathematical form but also on how they represent fiber recruitment, anisotropy, and the interaction between the ground matrix and collagen fiber network. Models that allow a smoother coupling between isotropic and anisotropic effects are more suitable for describing the biaxial mechanical behavior of porcine pericardium.

Under physiological conditions, soft tissue is subjected to shear forces, which affect the aortic valve. The ability to capture shearing effects and fiber dispersion was added to the Holzapfel (2015) model, which comes into effect when fibers are activated (Holzapfel and Ogden, 2015). Fiber dispersion in this model is classified as in-plane and out-of-plane fiber dispersion. The Gasser (2006) model used to characterize porcine pericardium in this study is a modification of the Holzapfel (2000) model by adding fiber dispersion κ, which describes fiber alignment in the material and adds the effects of shear on the material (Gasser et al., 2006). The strain energy function of the polynomial anisotropic model comprises an isotropic part represented by the first and second invariants and an anisotropic part represented by the two fiber stretch squares. It does not represent any shear effect, fiber dispersion, or weighting function. The transversely isotropic model takes the format of the Gasser (2006) model and changes the isotropic part of the model by replacing it with the Arruda–Boyce isotropic definition, which employs the first invariant and the effective matrix stiffness. The anisotropic 8-chain model takes a series of Arruda–Boyce models and describes the isotropic part in the same way as Arruda–Boyce does, namely, by elastin chain stretch, which is dependent on the first invariant, whereas the anisotropic part is defined by the fiber chain stretch, which is dependent on the fourth invariant. The detailed formulation of the anisotropic 8-chain model is provided in Supplementary Material S1.

The results obtained from the experiments show that porcine pericardium is anisotropic since the circumferential and radial responses of the material when subjected to equibiaxial tensile testing are different after 500 mN. At the initial stages of material deformation, the pericardium response is coupled in both directions, although the circumferential direction resists deformation better than the radial direction. The use of LM and GA to find the optimum material parameters is a hybrid approach intended to eliminate the downside of one optimizer by replacing it with another when it becomes trapped in local minima or local maxima (Pandey et al., 2024). The LM algorithm converges more quickly but sometimes gets stuck in local minima (Hassan et al., 2016). When this occurred, the parameters obtained were transferred to GA for continuation. Both optimizers may give different parameters when starting from the same initial points.

After finding material parameters for each of the nine experiments using all the above-mentioned constitutive models, the average parameters for each model were used to fit the average circumferential and radial responses of the experimental results. Through visual inspection, the Holzapfel (2005) and four-fiber-family models produced good results, but the transversely isotropic model and the anisotropic 8-chain model fitted better than the remaining models, and this was done using independent average results. This finding suggests that the use of the coefficient of determination to decide which constitutive model best fits the data is not accurate enough for identifying material parameters. Future studies should focus on using a more accurate evaluation index and using machine learning to find optimum parameters. For further evaluation of these constitutive models, an evaluation index based on average coefficients of determination was used, and it was found that the Fung, Holzapfel (2005), and four-fiber-family models achieved good evaluation index results.

From a clinical standpoint, EOA and pressure gradients are directly related to valve obstruction severity and are routinely used for grading stenosis, whereas WSS-based measures provide complementary insight into near-leaflet shear environments that may contribute to adverse biological responses, including remodeling, thrombus-related phenomena, and structural valve deterioration. Therefore, even when global metrics indicate normal function, differences in leaflet constitutive assumptions can still alter local shear exposure during ejection and the closure transient, which may be relevant for long-term durability considerations. In the present study, both material models yield global hemodynamic values consistent with normal valve performance, while differences are most pronounced in the closure-phase WSS peaks, supporting the need for experimentally grounded material parameterization when using FSI for bioprosthetic valve assessment.

### 4.2 FSI simulation

The computational results indicate that both selected anisotropic material models reproduce a functionally unobstructed bioprosthetic valve response under the prescribed loading conditions. This is supported not only by the absolute values of peak velocity and pressure gradient but also by the consistency between orifice opening, jet formation, and pressure recovery. From the standpoint of prosthetic valve hemodynamics, this combination is important because elevated transvalvular gradients are usually associated with insufficient effective opening, increased flow acceleration, or prosthesis–patient mismatch, whereas adequate EOA reflects the ability of the valve to provide a sufficiently large effective flow area (Hahn and Pibarot, 2024). In this context, the low-pressure gradients and the EOA values of 2.15–2.38 cm^2^ obtained in the present model support the interpretation that neither material model produced a stenosis-like flow obstruction.

The relationship between geometric opening and effective hydrodynamic performance should be interpreted with caution since GOA and EOA describe different aspects of valve function. GOA characterizes the anatomical opening formed by the leaflets, whereas EOA reflects the effective cross-sectional area of the jet after flow contraction and energy losses. Whiting et al. (2022) showed that the geometric orifice area and opening pressure obtained from computational valve models can be used as the predictors of the experimentally measured effective orifice area and transvalvular pressure decrease. Therefore, the present comparison of GOA, EOA, and pressure gradients provides complementary information: similar global values indicate comparable macroscopic opening, whereas differences in jet structure and pressure recovery reveal model-dependent hydrodynamic behavior.

The differences between the Holzapfel (2005) and four-fiber-family models were more pronounced in local flow organization than in global valve performance. The four-fiber-family model produced a slightly more extended high-velocity jet core, while the Holzapfel (2005) model generated more pronounced WSS peaks during closure. This suggests that the constitutive model affects not only the maximum leaflet opening but also the transient shape of the orifice, the contraction of the systolic jet, and the redistribution of momentum downstream of the valve. A similar sensitivity of hemodynamic indices to leaflet configuration was reported by Kim et al. (2024), who showed in two-way FSI simulations of bioprosthetic aortic valves that design-related changes in leaflet and strut geometry affected velocity, EOA, stroke volume, leakage volume, and leaflet stress. Thus, even relatively small differences in leaflet deformation may alter both mechanical loading and local flow characteristics.

The observed WSS patterns further demonstrate that local hemodynamic indices are more sensitive to material-model choice than global quantities such as EOA or ΔPmax. In both models, elevated WSS was concentrated near the free edges and commissures, which is consistent with the expected localization of high tangential velocity gradients in regions exposed to accelerated jet flow and rapid leaflet motion. Recent modeling studies also indicate that changes in leaflet geometry and coaptation affect transvalvular pressure gradients, jet characteristics, and WSS distributions (Mutlu et al., 2026). In the present study, the stronger closure-phase WSS peaks in the Holzapfel (2005) model may be associated with differences in leaflet stiffness, contact dynamics, and the rate of orifice reduction during late systole. Therefore, WSS should be interpreted not only as a flow descriptor but also as an indicator of localized leaflet loading that may be relevant for long-term durability assessment.

The formation of secondary flow structures and vortical regions in the aortic root and ascending aorta also supports the need for a coupled FSI formulation. Valve leaflets do not simply define a static flow obstruction; their motion continuously modifies the jet direction, local acceleration, recirculation zones, and pressure recovery. Fumagalli et al. (2025) showed that FSI-type formulations can reproduce leaflet motion, flow in the ascending aorta, and the pressure jump across the valve, emphasizing the importance of including valve dynamics in hemodynamic analysis. Patient-specific FSI studies have also demonstrated that changes in valve function may affect EOA, leaflet stress, and viscous dissipation in the ascending aorta (Govindarajan et al., 2025). These findings are consistent with the present results, where the choice of the leaflet material model influenced not only deformation but also downstream jet organization, WSS distribution, pressure recovery, and vortex formation.

The possible influence of the surrounding aortic tissue properties should also be considered. Wagenseil (2011) showed that aortic wall mechanics substantially depend on extracellular matrix organization, elastin content, wall stiffness, and regional material properties. In addition, Wu et al. (2025) demonstrated that biomechanical characteristics of the aortic wall, including circumferential and axial incremental moduli, are associated with changes in aortic diameter during aneurysm progression. These factors may alter the mechanical environment of the aortic wall and affect local hemodynamic characteristics in coupled simulations. In the present model, the aortic root was treated as a fixed boundary, and the bioprosthetic frame was rigidly fixed within it. This formulation was chosen to provide a controlled computational setup and to focus the analysis on the influence of leaflet constitutive behavior. However, aortic root compliance and surrounding tissue deformation may affect pressure recovery, WSS, vortical structures, and leaflet–root interaction. We believe that this study of hemodynamic sensitivity could be conducted as a separate future study, similar to the work of Cai et al. (2019) on the mitral valve. Nevertheless, we have acknowledged this limitation in the text.

A limitation of the present study is the absence of a formal sensitivity analysis quantifying how variations in the identified HGO and four-fiber material parameters affect key hemodynamic outputs. Given the demonstrated sensitivity of arterial and valve FSI models to constitutive parameters, we recognize that this is essential for interpreting the physiological relevance of our results (Balzani et al., 2023).

Another important limitation is the modeling of the aortic root and ascending aorta as rigid structures. Although this assumption enabled a controlled comparison of leaflet-constitutive models by eliminating confounding variables associated with wall compliance, it does not reflect physiological reality. As demonstrated by Wagenseil (2011) in mouse aorta models, vessel wall composition and mechanical properties fundamentally alter the local mechanical environment. In the context of aortic valve FSI, compliant walls allow pressure wave propagation, energy dissipation, and wall motion that can affect leaflet kinematics, particularly during deceleration and closure. Díaz et al. (2021) showed that multilayer models of the aorta incorporating layer-specific residual stresses (intima, media, and adventitia) are necessary to accurately reproduce biaxial mechanical responses and internal pressure behavior, with *R* ^2^ values increasing from 0.70 to 0.98 when residual stresses are included. Future work will incorporate a hyperelastic, multilayer aortic wall model with fiber-reinforced layers (using the same HGO framework applied to the leaflets) and, ideally, subject-specific residual stress distributions. We anticipate that this refinement would reduce peak WSS values and alter the vortical structures in the sinuses, bringing the predictions closer to *in vivo* measurements. However, the rigid wall assumption does not invalidate the primary conclusion of this study—namely, that leaflet constitutive model choice affects local hemodynamic indices (WSS and vorticity) even when global metrics (EOA and ΔP) remain similar—as both material models were compared under identical rigid-wall boundary conditions.

Overall, the comparison shows that both experimentally identified anisotropic models provide physiologically acceptable global valve performance, but they are not equivalent in terms of local hemodynamic loading. The four-fiber-family model appears to produce a more coherent and extended systolic jet, whereas the Holzapfel (2005) model leads to stronger localized shear peaks during closure. This distinction is important because global indices may indicate normal valve function, while local WSS and vortical structures still differ substantially. Therefore, the main implication of the computational analysis is that experimentally informed constitutive models should be used not only to reproduce leaflet deformation but also to obtain reliable local hemodynamic indicators relevant to bioprosthetic valve durability.

### 4.3 Study limitations

We acknowledge an important methodological nuance raised regarding the use of engineering (nominal) stress and strain measures in the initial presentation of our experimental data (Equations 2, 3). Although hyperelastic constitutive models are fundamentally formulated in terms of work-conjugate finite strain measures—specifically the Green–Lagrange strain tensor **E** and the second Piola–Kirchhoff stress tensor **S**, we reported engineering values. The nature of the limitation is that engineering stress and engineering strain are valid only for infinitesimal deformations. Soft biological tissues, including porcine pericardium, routinely undergo finite deformations (10%–30% strain or higher) during physiological loading. Consequently, engineering measures do not correctly represent the true thermodynamic state of the material and are not directly compatible with hyperelastic strain-energy functions. Nevertheless, there are some approaches to re-evaluate obtained data through large deformation relations. In addition, it should be noted that the resultant strain was less than 10%; therefore, we found that the difference between the engineering and true strains was negligible.

When using an optimizer to obtain optimum material parameters, arbitrary initial parameters can affect the optimizer’s efficiency, and the results depend on initial conditions. Only eight popularly used anisotropic constitutive models were selected, and two novel models were selected, which limits our selection to only these 10 constitutive models. The effects of gripping methods, strain rate, preconditioning cycles, and test specimen sizes could have effects on the obtained results; however, these factors were not investigated, although the results obtained are accurate, and a finite element method model can be developed using these parameters.

Several simplifying assumptions were introduced in the computational FSI model to provide a controlled and numerically stable framework for comparing the influence of leaflet-constitutive descriptions. The geometry of the aortic root, ascending aorta, and leaflets was idealized and parameterized, which reduced anatomical variability and allowed the effect of the material model to be isolated from patient-specific geometric factors. Similarly, the aortic root and ascending aorta were treated as rigid walls to focus the analysis on leaflet deformation and its direct impact on valve kinematics, pressure gradients, WSS, and vortical structures. Although this assumption neglects aortic wall compliance, it provides a reproducible setup for comparing the Holzapfel (2005) and four-fiber-family models under identical boundary conditions. In addition, the surrounding aortic tissues were not explicitly included as deformable domains. The bioprosthetic frame was rigidly fixed within the aortic root, and the aortic root wall was treated as a fixed boundary. Although these assumptions enabled a controlled and reproducible comparison of leaflet material models, aortic root compliance and surrounding tissue deformation may influence local pressure recovery, WSS, and vortical structures. Future studies should, therefore, incorporate deformable aortic walls and surrounding tissue models to assess their impact on valve biomechanics and hemodynamics.

Blood flow was modeled under the laminar flow assumption, although the average Reynolds number was 3,685 and therefore lies within the transitional range. This choice reduced the number of additional modeling parameters and allowed the observed differences in velocity, WSS, pressure gradients, and vortex structures to be attributed primarily to the leaflet material behavior rather than to turbulence-model effects. Therefore, the local flow quantities should be interpreted as comparative indicators between the selected material models rather than as a complete description of transitional or turbulent flow features. The Newtonian blood assumption and the penalty-based leaflet contact formulation with a minimum clearance were also used to improve numerical robustness and preserve ALE mesh integrity during valve closure. These assumptions may affect near-leaflet flow and closure dynamics, but they enabled stable coupled simulations over the cardiac cycle.

### 4.4 Concluding remarks

The Landrace pig’s pericardium is an anisotropic hyperelastic material that correlates with human aortic valve leaflets and justifies why it is used as a material for the design of bioprosthetic aortic valves. A blend of the LM and GA methods produced reliable parameters for all 10 models, and these parameters can be embedded into FEA simulations to compare simulation results with experimental results. The Fung, Holzapfel (2005), and four-fiber family constitutive models captured the material response of porcine pericardium, which was subjected to equibiaxial tensile tests, according to the evaluation index used. However, through visual inspection, it could be observed that the transversely isotropic and anisotropic 8-chain models fitted the independent averaged data better than the remaining models, as shown in Figure 6. The use of bio-rakes for biaxial tensile tests reduces shear effects; hence, both the Holzapfel (2015) and Gasser (2006) models used in this study did not perform well. Based on the average coefficients of determination, it can be concluded that some of the chosen constitutive models do not adequately predict material responses; these models are Holzapfel (2000), Holzapfel (2015), Gasser 2006, Polynomial anisotropy, and Choi–Vito constitutive models. The Fung, Holzapfel (2005), and four-fiber-family models produced good responses according to the evaluation index used. The results shown in Figure 2 suggest that the evaluation index used is misleading, and this could be because it only uses the coefficient of determination or simply because the predicted parameters are not good for independent experimental results. The response of the material differs depending on the type of loading to which it is subjected. It is recommended to capture all material responses and obtain a constitutive model that best predicts the response of the material by performing uniaxial tension, shear, and biaxial tests and characterizing the pericardium under these three loading modes. Initial guesses can lead to different solutions and can be considered a gap; the solution should be independent of the initial guesses when optimizers are used.

## Data Availability

The original contributions presented in the study are included in the article/Supplementary Material; further inquiries can be directed to the corresponding author.
