# Supplementary material for: Anisotropic hyperelastic properties of porcine pericardium under equibiaxial loading: implications for aortic valve design
Source: Front Bioeng Biotechnol. 2026 Aug 26;14:1840657. doi: 10.3389/fbioe.2026.1840657 (PMC13553837; doi:10.3389/fbioe.2026.1840657)
Supplement: Supplementary file 1 [file Supplementaryfile3.docx]

**Supplementary material 2. Parameters of constitutive equations.**

The use of Fung’s constitutive model to fit the experimental data of the pericardium produced results tabulated in Table 1. An average for the result was calculated with its standard deviation, and the obtained parameters assure numerical stability when employing Finite element analysis for simulation, since they adhere to the following conditions: $A_{1}A_{2}>{A_{4}}^{2}, A_{2}A_{3}>{A_{23}}^{2}, \mathrm{and}A_{1}A_{3}>{A_{13}}^{2}$.

Table S1 Fung's constitutive model optimized material parameters

|  | Exp_1 | Exp_2 | Exp_3 | Exp_4 | Exp_5 | Exp_6 | Exp_7 | Exp_8 | Exp_9 | Average | STD |
| --- | --- | --- | --- | --- | --- | --- | --- | --- | --- | --- | --- |
| c | 0.03 | 0.02 | 0.04 | 0.01 | 0.09 | 0.04 | 0.04 | 0.02 | 0.03 | 0.04 | 0.023 |
| b1 | 84.91 | 123.84 | 80.11 | 94.59 | 28.48 | 35.94 | 35.07 | 83.04 | 55.71 | 69.08 | 32.208 |
| b2 | 62.26 | 109.01 | 38.58 | 47.89 | 23.11 | 66.39 | 73.54 | 98.12 | 94.46 | 68.15 | 28.810 |
| b3 | 0.31 | 0.46 | 0.22 | 0.15 | 0.17 | 0.66 | 0.08 | 0.30 | 0.40 | 0.31 | 0.180 |
| b4 | 3.50 | 4.16 | 5.41 | 1.23 | 1.83 | 7.43 | 1.19 | 0.97 | 2.13 | 3.09 | 2.225 |
| b5 | 0.39 | 0.76 | 0.34 | 0.70 | 0.15 | 0.23 | 1.5 5 | 1.03 | 1.20 | 0.70 | 0.479 |
| b6 | 2.25 | 0.35 | 1.13 | 0.39 | 1.58 | 1.23 | 0.54 | 0.29 | 0.33 | 0.90 | 0.692 |
| SSE | 0.11 | 0.10 | 0.09 | 0.01 | 0.15 | 0.11 | 0.13 | 0.08 | 0.14 | 0.10 | 0.041 |
| SAE | 4.13 | 4.29 | 1.97 | 0.64 | 4.54 | 4.05 | 4.35 | 3.80 | 4.67 | 3.61 | 1.370 |
| NE | 0.03 | 0.03 | 0.13 | 0.09 | 0.04 | 0.03 | 0.04 | 0.03 | 0.04 | 0.05 | 0.035 |
| NRMSE | 0.05 | 0.04 | 0.17 | 0.11 | 0.05 | 0.04 | 0.05 | 0.04 | 0.06 | 0.07 | 0.044 |
| r | 0.99 | 0.99 | 0.97 | 0.99 | 0.99 | 0.99 | 0.99 | 0.99 | 0.99 | 0.99 | 0.007 |
| R2 | 0.98 | 0.98 | 0.94 | 0.98 | 0.98 | 0.98 | 0.98 | 0.99 | 0.98 | 0.98 | 0.014 |

To obtain material parameters using Choi-Vito’s constitutive model $b_{i}$ parameters were chosen to be anything above zero to obtain parameters with physical sense and numerical stability for FEA. Choi-Vito fitted parameters are shown in Table 2 and results average with standard deviations and different errors with coefficients of correlation and coefficients of determination.

|  | Exp_1 | Exp_2 | Exp_3 | Exp_4 | Exp_5 | Exp_6 | Exp_7 | Exp_8 | Exp_9 | Average | STD |
| --- | --- | --- | --- | --- | --- | --- | --- | --- | --- | --- | --- |
| b0 | 0.007 | 0.01 | 0.00 | 0.002 | 0.01 | 0.01 | 0.01 | 0.004 | 0.005 | 0.01 | 0.004 |
| b1 | 262.28 | 287.17 | 458.13 | 296.64 | 99.25 | 155.60 | 144.00 | 290.01 | 223.02 | 246.23 | 150.797 |
| b2 | 193.80 | 253.57 | 252.79 | 97.19 | 89.47 | 347.56 | 320.78 | 344.06 | 410.05 | 256.59 | 107.456 |
| b3 | 114.80 | 230.57 | 41.12 | 160.58 | 65.58 | 122.15 | 114.79 | 178.30 | 125.18 | 128.12 | 62.710 |
| SS | 0.10 | 0.16 | 0.03 | 0.003 | 0.15 | 0.08 | 0.12 | 0.07 | 0.12 | 0.09 | 0.052 |
| SA | 3.74 | 4.85 | 0.91 | 0.39 | 4.49 | 3.63 | 4.52 | 3.61 | 4.43 | 3.40 | 1.625 |
| NE | 0.03 | 0.04 | 0.07 | 0.07 | 0.04 | 0.03 | 0.04 | 0.03 | 0.04 | 0.04 | 0.017 |
| NRMSE | 0.04 | 0.05 | 0.11 | 0.09 | 0.05 | 0.04 | 0.05 | 0.04 | 0.05 | 0.06 | 0.024 |
| r | 0.99 | 0.99 | 0.99 | 0.99 | 0.99 | 0.99 | 0.99 | 0.99 | 0.99 | 0.99 | 0.002 |
| R2 | 0.98 | 0.97 | 0.98 | 0.99 | 0.98 | 0.99 | 0.98 | 0.99 | 0.98 | 0.98 | 0.006 |

Table S2 Choi-Vito constitutive model optimized material parameters

To obtain numerical stability and physically meaningful parameters $a_{i},b_{k}\mathrm{and} c_{j}$ had to be greater than zero and angles for fibers were initialized at 30 degrees arbitrarily, the angles decision depends on visual inspection of the fibers and the Levenberg Marquardt and Genetic algorithms do not significantly change the initialized angle when searching for material parameters. Table 3 shows material parameters obtained from using polynomial models.

Table S3 Polynomial (anisotropic) constitutive model optimized material parameters

|  | Exp_1 | Exp_2 | Exp_3 | Exp_4 | Exp_5 | Exp_6 | Exp_7 | Exp_8 | Exp_9 | Average | STD |
| --- | --- | --- | --- | --- | --- | --- | --- | --- | --- | --- | --- |
| a1 | 0.04 | 0.07 | 0.001 | 0.017 | 0.19 | 0.13 | 0.13 | 0.24 | 0.16 | 0.11 | 0.082 |
| a2 | 1.40 | 5.78 | 3.21 | 0.007 | 0.03 | 0.41 | 0.41 | 0.43 | 0.45 | 1.35 | 1.938 |
| a3 | 0.52 | 1.73 | 2.45 | 3.394 | 3.34 | 3.68 | 3.68 | 3.68 | 3.68 | 2.91 | 1.124 |
| b1 | 1.09 | 1.04 | 0.13 | 12.627 | 8.65 | 8.65 | 8.65 | 8.65 | 8.65 | 6.46 | 4.476 |
| b2 | 0.80 | 5.09 | 0.05 | 0.701 | 1.81 | 1.81 | 1.81 | 1.81 | 1.81 | 1.74 | 1.417 |
| b3 | 0.63 | 0.17 | 0.39 | 0.138 | 0.06 | 0.06 | 0.06 | 0.06 | 0.06 | 0.18 | 0.200 |
| c2 | 0.06 | 0.00 | 0.11 | 0.001 | 0.02 | 0.00 | 0.00 | 0.09 | 0.08 | 0.04 | 0.045 |
| c3 | 0.22 | 0.00 | 0.11 | 0.113 | 0.01 | 0.41 | 0.40 | 0.44 | 0.46 | 0.24 | 0.189 |
| c4 | 3.12 | 4.18 | 0.60 | 2.060 | 2.00 | 3.03 | 3.03 | 3.04 | 3.04 | 2.68 | 1.008 |
| c5 | 5.03 | 0.35 | 1.08 | 0.805 | 0.16 | 0.44 | 0.44 | 0.44 | 0.44 | 1.02 | 1.527 |
| c6 | 30.83 | 1.85 | 11.95 | 1.164 | 1.55 | 1.62 | 1.62 | 1.62 | 1.62 | 5.98 | 9.931 |
| phi | 22.76 | 27.59 | 40.57 | 25.598 | 29.05 | 24.24 | 24.22 | 24.33 | 24.27 | 26.96 | 5.461 |
| SS | 0.11 | 0.06 | 0.004 | 0.009 | 0.16 | 0.08 | 0.28 | 0.21 | 0.22 | 0.13 | 0.098 |
| SA | 3.98 | 3.44 | 0.36 | 0.66 | 4.73 | 3.42 | 6.28 | 5.03 | 5.12 | 3.67 | 2.004 |
| NE | 0.03 | 0.03 | 0.04 | 0.08 | 0.04 | 0.03 | 0.05 | 0.04 | 0.04 | 0.04 | 0.016 |
| NRMSE | 0.05 | 0.03 | 0.05 | 0.10 | 0.06 | 0.04 | 0.07 | 0.06 | 0.07 | 0.06 | 0.020 |
| r | 0.99 | 0.99 | 1.00 | 0.99 | 0.99 | 0.99 | 0.98 | 0.99 | 0.99 | 0.99 | 0.005 |
| R2 | 0.98 | 0.99 | 1.00 | 0.99 | 0.97 | 0.99 | 0.95 | 0.96 | 0.96 | 0.98 | 0.017 |

The strain energy for the Holzapfel 2000 model has two parts, which are the isotropic part and the anisotropic part. The isotropic part is represented by mu, which should always be greater than zero. Values for $k_{1}\mathrm{and} k_{2}$ were chosen to be any value above zero, and the threshold for the angle was 0 to 90 degrees. Holzapfel's 2000 material parameters are presented in Table 4 with average results, errors, and coefficients of correlation and determination included.

Table S4 Holzapfel (2000) constitutive model optimized material parameters

|  | Exp_1 | Exp_2 | Exp_3 | Exp_4 | Exp_5 | Exp_6 | Exp_7 | Exp_8 | Exp_9 | Average | STD |
| --- | --- | --- | --- | --- | --- | --- | --- | --- | --- | --- | --- |
| mu | 0.001 | 0.001 | 0.001 | 0.01 | 0.001 | 0.12 | 0.001 | 0.001 | 0.21 | 0.04 | 0.075 |
| k1 | 0.32 | 0.63 | 0.46 | 0.15 | 0.56 | 0.44 | 0.61 | 0.55 | 0.36 | 0.45 | 0.156 |
| k2 | 61.20 | 57.00 | 45.59 | 37.94 | 13.92 | 27.43 | 19.92 | 41.32 | 41.64 | 38.44 | 15.803 |
| phi | 32.14 | 44.75 | 32.09 | 47.68 | 74.61 | 58.86 | 41.67 | 41.63 | 25.97 | 44.38 | 14.955 |
| SS | 0.02 | 0.11 | 0.02 | 0.00 | 0.15 | 0.23 | 0.34 | 0.09 | 0.24 | 0.13 | 0.117 |
| SA | 0.84 | 4.46 | 0.92 | 0.42 | 4.56 | 5.91 | 6.88 | 4.03 | 6.09 | 3.79 | 2.468 |
| NE | 0.08 | 0.03 | 0.09 | 0.07 | 0.04 | 0.05 | 0.06 | 0.03 | 0.05 | 0.06 | 0.021 |
| NRMSE | 0.10 | 0.04 | 0.11 | 0.09 | 0.06 | 0.07 | 0.08 | 0.04 | 0.07 | 0.07 | 0.024 |
| r | 0.99 | 0.99 | 0.99 | 0.99 | 0.99 | 0.98 | 0.98 | 0.99 | 0.99 | 0.99 | 0.004 |
| R2 | 0.99 | 0.98 | 0.98 | 0.99 | 0.98 | 0.96 | 0.94 | 0.98 | 0.96 | 0.97 | 0.017 |

The difference in formulation of the Holzapfel 2005 and the preceding model is the weight function rho, which is an element of 0 to 1, with zero being isotropic and one being fully anisotropic. Table 5 shows material parameters obtained from using the Holzapfel 2005 strain energy density function.

Table S5 Holzapfel (2005) constitutive model optimized material parameters

|  | Exp_1 | Exp_2 | Exp_3 | Exp_4 | Exp_5 | Exp_6 | Exp_7 | Exp_8 | Exp_9 | Average | STD |
| --- | --- | --- | --- | --- | --- | --- | --- | --- | --- | --- | --- |
| mu | 0.001 | 0.003 | 0.001 | 0.03 | 0.12 | 0.03 | 0.07 | 0.06 | 0.001 | 0.04 | 0.041 |
| k1 | 3.63 | 7.69 | 3.67 | 0.30 | 1.73 | 2.87 | 2.47 | 4.83 | 3.81 | 3.45 | 2.070 |
| k2 | 55.80 | 7.02 | 16.32 | 70.94 | 3.79 | 1.84 | 2.97 | 1.90 | 1.71 | 18.03 | 26.383 |
| phi | 34.65 | 21.61 | 25.22 | 28.55 | 43.68 | 29.97 | 29.97 | 26.70 | 20.00 | 28.93 | 7.112 |
| rho | 0.03 | 0.01 | 0.07 | 0.30 | 0.01 | 0.07 | 0.09 | 0.01 | 0.06 | 0.07 | 0.091 |
| SS | 0.01 | 0.06 | 0.005 | 0.01 | 0.12 | 0.10 | 0.17 | 0.05 | 0.10 | 0.07 | 0.057 |
| SA | 0.54 | 3.29 | 0.44 | 0.54 | 3.84 | 3.74 | 4.93 | 2.85 | 4.03 | 2.69 | 1.730 |
| NE | 0.05 | 0.03 | 0.04 | 0.09 | 0.03 | 0.03 | 0.04 | 0.02 | 0.03 | 0.04 | 0.021 |
| NRMSE | 0.07 | 0.03 | 0.05 | 0.11 | 0.05 | 0.04 | 0.06 | 0.03 | 0.05 | 0.05 | 0.025 |
| r | 1.00 | 0.99 | 1.00 | 0.99 | 0.99 | 0.99 | 0.99 | 1.00 | 0.99 | 0.99 | 0.005 |
| R2 | 0.99 | 0.99 | 1.00 | 0.98 | 0.98 | 0.98 | 0.97 | 0.99 | 0.98 | 0.99 | 0.009 |

The difference between the Four-fiber family and the Holzapfel 2000 model is the inclusion of other fiber families, which are represented by $c_{1i}$ and $c_{2i}$ representing four fiber stiffnesses and four nonlinearities of parameter, respectively. Table 6 contains the material parameters obtained.

Table S6 Four Fibers Family constitutive model optimized material parameters

|  | Exp_1 | Exp_2 | Exp_3 | Exp_4 | Exp_5 | Exp_6 | Exp_7 | Exp_8 | Exp_9 | Average | STD |
| --- | --- | --- | --- | --- | --- | --- | --- | --- | --- | --- | --- |
| c | 0.001 | 0.08 | 0.001 | 0.01 | 0.001 | 0.06 | 0.001 | 0.001 | 0.04 | 0.02 | 0.031 |
| c1_1 | 0.32 | 0.97 | 0.39 | 0.32 | 1.08 | 0.51 | 0.41 | 0.51 | 0.68 | 0.58 | 0.279 |
| c2_1 | 10.16 | 50.49 | 43.37 | 31.66 | 9.31 | 84.50 | 74.73 | 83.87 | 83.19 | 52.36 | 30.870 |
| c1_2 | 0.69 | 0.81 | 0.90 | 0.33 | 0.97 | 0.61 | 0.50 | 1.13 | 0.78 | 0.75 | 0.245 |
| c2_2 | 72.56 | 89.83 | 68.10 | 38.88 | 21.65 | 8.49 | 9.21 | 32.18 | 22.53 | 40.38 | 29.531 |
| c1_34 | 0.37 | 0.21 | 0.23 | 0.00 | 0.08 | 0.14 | 0.48 | 0.11 | 0.12 | 0.19 | 0.150 |
| c2_34 | 28.13 | 8.56 | 13.65 | 33.62 | 3.45 | 49.69 | 28.03 | 14.44 | 21.34 | 22.32 | 14.260 |
| phiO | 36.04 | 20.32 | 22.85 | 20.00 | 23.42 | 36.93 | 29.12 | 23.86 | 20.00 | 25.84 | 6.663 |
| SS | 0.08 | 0.11 | 0.01 | 0.004 | 0.15 | 0.06 | 0.11 | 0.08 | 0.11 | 0.08 | 0.048 |
| SA | 3.45 | 3.98 | 0.60 | 0.38 | 4.50 | 3.01 | 3.79 | 3.86 | 4.06 | 3.07 | 1.520 |
| NE | 0.03 | 0.03 | 0.06 | 0.08 | 0.04 | 0.02 | 0.03 | 0.03 | 0.03 | 0.04 | 0.019 |
| NRMSE | 0.04 | 0.04 | 0.07 | 0.10 | 0.05 | 0.03 | 0.05 | 0.04 | 0.05 | 0.05 | 0.021 |
| r | 0.99 | 0.99 | 1.00 | 0.99 | 0.99 | 1.00 | 0.99 | 0.99 | 0.99 | 0.99 | 0.004 |
| R2 | 0.99 | 0.98 | 0.99 | 0.98 | 0.98 | 0.99 | 0.98 | 0.99 | 0.98 | 0.98 | 0.005 |

Gasser's 2006 material parameters are given in Table 7, from Holzapfel’s 2000; Gasser included the fiber dispersion parameter $\kappa\in(0,\frac{1}{3})$ and the fibers and shear coupling parameter,, which is $\gamma\geq0$ (Holzapfel et al., 2019).

Table S7 Gasser (2006) constitutive model optimized the material parameters

|  | Exp_1 | Exp_2 | Exp_3 | Exp_4 | Exp_5 | Exp_6 | Exp_7 | Exp_8 | Exp_9 | Average | STD |
| --- | --- | --- | --- | --- | --- | --- | --- | --- | --- | --- | --- |
| c10 | 0.001 | 0.001 | 0.001 | 0.034 | 0.001 | 0.001 | 0.001 | 0.00 | 0.001 | 0.005 | 0.011 |
| k1 | 2.15 | 2.85 | 2.29 | 0.09 | 2.265 | 2.29 | 2.09 | 2.58 | 2.05 | 2.07 | 0.784 |
| k2 | 122.97 | 178.74 | 133.66 | 46.47 | 121.803 | 147.13 | 141.41 | 197.48 | 240.76 | 147.83 | 54.623 |
| gamma | 0.77 | 0.76 | 0.64 | 0.52 | 0.783 | 0.95 | 1.00 | 0.83 | 0.92 | 0.80 | 0.152 |
| kappa | 0.19 | 0.19 | 0.19 | 0.00 | 0.221 | 0.21 | 0.20 | 0.21 | 0.21 | 0.18 | 0.069 |
| SS | 0.08 | 0.05 | 0.06 | 9.87 | 0.13 | 0.13 | 0.23 | 0.05 | 0.12 | 1.19 | 3.255 |
| SA | 3.55 | 3.21 | 1.55 | 16.73 | 4.15 | 4.16 | 5.28 | 2.85 | 4.05 | 5.06 | 4.497 |
| NE | 0.03 | 0.03 | 0.11 | 0.12 | 0.03 | 0.03 | 0.04 | 0.02 | 0.03 | 0.05 | 0.038 |
| NRMSE | 0.04 | 0.03 | 0.15 | 0.16 | 0.05 | 0.05 | 0.07 | 0.03 | 0.05 | 0.07 | 0.050 |
| r | 0.99 | 0.99 | 0.98 | 0.99 | 0.99 | 0.99 | 0.98 | 1.00 | 0.99 | 0.99 | 0.006 |
| R2 | 0.99 | 0.99 | 0.96 | 0.97 | 0.98 | 0.98 | 0.96 | 0.99 | 0.98 | 0.98 | 0.012 |

The Holzapfel 2015 model takes into consideration the shear effects when stretching a material, and that coupling is represented by $\gamma$ which is greater than or equal to zero, and when it is equal to zero, it means no coupling, and greater than zero means fibers are opposing shear (Holzapfel and Ogden, 2015) (Holzapfel and Ogden, 2015). The in-plane and out-of-plane fiber dispersion are introduced, which are $\kappa_{inp}\in(0,1)$ and $\kappa_{outp}\in(0,0.5)$ respectively. Holzapfel's 2015 fitting parameters are shown in Table S8.

Table S8 Holzapfel (2015) constitutive model optimized material parameters

|  | Exp_1 | Exp_2 | Exp_3 | Exp_4 | Exp_5 | Exp_6 | Exp_7 | Exp_8 | Exp_9 | Average | STD |
| --- | --- | --- | --- | --- | --- | --- | --- | --- | --- | --- | --- |
| Mu | 0.0010 | 0.0010 | 0.0010 | 0.0803 | 0.0010 | 0.0010 | 0.0010 | 0.0010 | 0.0010 | 0.0098 | 0.026 |
| k1 | 0.44 | 0.64 | 0.71 | 1.52 | 0.97 | 0.99 | 0.94 | 1.09 | 1.15 | 0.9388 | 0.316 |
| k2 | 41.44 | 56.56 | 40.49 | 332.36 | 19.25 | 33.37 | 53.94 | 67.66 | 47.83 | 76.988 | 96.781 |
| gamma | 0.76 | 0.39 | 0.37 | 0.16 | 0.79 | 0.47 | 0.37 | 0.64 | 0.35 | 0.4777 | 0.210 |
| kappa_ip | 0.34 | 0.48 | 0.36 | 0.36 | 0.59 | 0.55 | 0.61 | 0.52 | 0.54 | 0.4833 | 0.105 |
| kappa_OP | 0.50 | 0.50 | 0.48 | 0.36 | 0.46 | 0.46 | 0.45 | 0.45 | 0.45 | 0.4566 | 0.042 |
| SS | 0.14 | 0.11 | 0.07 | 0.01 | 0.14 | 0.21 | 0.13 | 0.09 | 0.21 | 0.1233 | 0.064 |
| SA | 4.66 | 4.51 | 1.73 | 0.50 | 4.55 | 5.39 | 2.93 | 3.88 | 5.37 | 3.72 | 1.684 |
| NE | 0.04 | 0.04 | 0.13 | 0.08 | 0.04 | 0.04 | 0.11 | 0.03 | 0.05 | 0.06 | 0.036 |
| NRMSE | 0.05 | 0.05 | 0.17 | 0.10 | 0.05 | 0.06 | 0.13 | 0.04 | 0.07 | 0.08 | 0.044 |
| R | 0.99 | 0.99 | 0.98 | 0.99 | 0.99 | 0.98 | 0.98 | 0.99 | 0.99 | 0.99 | 0.005 |
| R2 | 0.97 | 0.98 | 0.95 | 0.99 | 0.98 | 0.96 | 0.96 | 0.98 | 0.97 | 0.97 | 0.013 |

Fig. 8 shows a plot of the average experimental results fitted with ten hyperelastic anisotropic constitutive models, and the average material parameters from all ten constitutive models were used to fit on averaged independent experimental data. Through virtual inspection on Fig. 8 (a), the Choi-Vito model does not do a good job in capturing the circumferential and axial strains, the Holzapfel (2015) shown in Fig. 8 (c). The model does not fit well in both directions, the Gasser (2006) model in Fig. 8 (d) shows good results in capturing circumferential deformation but lacks precision in the axial direction.

Holzapfel (2000), Fung’s, and the Polynomial model shown in Fig. 8 (f), (h), and (g) do not perform well in predicting material response in both directions. Both Holzapfel (2005) and the Four-fibre family models shown in Fig. 8 (b) and (e) show a superior response in both directions.

Table S9. Below shows the performance of the anisotropic 8-chain model on experimental data and on averaged experimental data. The obtained coefficient of determination was averaged at 87percent, the coefficient of correlation was averaged at 93 percent, and other judgmental error parameters were at desirable thresholds as seen in Table S9.

Table S9. Anisotropic 8 Chain model results after fitting to biaxial tensile test data.

|  | Exp_1 | Exp_2 | Exp_3 | Exp_4 | Exp_5 | Exp_6 | Exp_7 | Exp_8 | Exp_9 | Average | STD |
| --- | --- | --- | --- | --- | --- | --- | --- | --- | --- | --- | --- |
| mu_iso | 0.01 | 0.01 | 0.01 | 0.01 | 0.01 | 0.01 | 0.01 | 0.01 | 0.01 | 0.01 | 0.00 |
| N_iso | 1.02 | 1.03 | 1.04 | 1.04 | 1.04 | 1.04 | 1.03 | 1.03 | 1.03 | 1.03 | 0.01 |
| mu_aniso | 5.E+07 | 1.E+08 | 5.E+07 | 4.E-01 | 2.E+08 | 2.E+08 | 2.E+07 | 2.E+08 | 5.E+06 | 9.E+07 | 8.E+07 |
| N_aniso | 2.E+06 | 3.E+06 | 7.E+05 | 5.E+01 | 4.E+06 | 5.E+06 | 4.E+05 | 4.E+06 | 1.E+05 | 2.E+06 | 2.E+06 |
| kappa | 0.17 | 0.17 | 0.17 | 0.33 | 0.17 | 0.17 | 0.17 | 0.17 | 0.17 | 0.18 | 0.05 |
| SSE | 0.13 | 0.49 | 0.32 | 0.05 | 0.64 | 0.79 | 0.27 | 0.12 | 0.41 | 0.36 | 0.25 |
| SA | 3.36 | 6.70 | 4.51 | 1.75 | 7.65 | 8.02 | 4.76 | 3.08 | 6.27 | 5.12 | 2.17 |
| NE | 0.12 | 0.27 | 0.39 | 0.08 | 0.33 | 0.36 | 0.20 | 0.12 | 0.32 | 0.24 | 0.12 |
| NRMSE | 0.06 | 0.12 | 0.12 | 0.04 | 0.14 | 0.13 | 0.10 | 0.06 | 0.12 | 0.10 | 0.04 |
| r | 0.99 | 0.92 | 0.87 | 0.99 | 0.90 | 0.87 | 0.96 | 0.99 | 0.93 | 0.93 | 0.05 |
| R2 | 0.97 | 0.84 | 0.75 | 0.98 | 0.81 | 0.75 | 0.92 | 0.97 | 0.86 | 0.87 | 0.09 |

The fitting results for the Transversely isotropic model are shown in Table S10. Where the coefficient of determination was averaged at 89 percent, and the correlation coefficient was averaged at 94 percent, error margins were at acceptable thresholds.

Table S10. Transverse isotropic model results after fitting to biaxial tensile test data.

|  | Exp_1 | Exp_2 | Exp_3 | Exp_4 | Exp_5 | Exp_6 | Exp_7 | Exp_8 | Exp_9 | Average | STD |
| --- | --- | --- | --- | --- | --- | --- | --- | --- | --- | --- | --- |
| mu_eff | 6.E-06 | 4.E-06 | 5.E-09 | 1.E-05 | 2.E-05 | 8.E-06 | 1.E-05 | 9.E-06 | 1.E-05 | 9.E-06 | 5.E-06 |
| N | 3.86 | 3.46 | 3.15 | 3.23 | 3.21 | 3.26 | 3.40 | 3.42 | 4.02 | 3.45 | 0.30 |
| k1 | 0.45 | 0.46 | 0.18 | 0.33 | 0.39 | 0.39 | 0.37 | 0.44 | 0.31 | 0.37 | 0.09 |
| k2 | 80.24 | 38.54 | 35.60 | 33.08 | 36.31 | 34.35 | 48.39 | 51.41 | 61.49 | 46.60 | 15.83 |
| kappa | 0.00 | 0.00 | 0.00 | 0.00 | 0.00 | 0.00 | 0.00 | 0.00 | 0.00 | 0.00 | 0.00 |
| SSE | 0.09 | 0.38 | 0.36 | 0.04 | 0.47 | 0.68 | 0.18 | 0.07 | 0.30 | 0.29 | 0.21 |
| SA | 2.75 | 5.98 | 4.82 | 1.54 | 6.41 | 7.34 | 3.88 | 2.35 | 5.33 | 4.49 | 1.99 |
| NE | 0.10 | 0.25 | 0.42 | 0.07 | 0.28 | 0.33 | 0.17 | 0.09 | 0.27 | 0.22 | 0.12 |
| NRMSE | 0.05 | 0.11 | 0.13 | 0.04 | 0.12 | 0.12 | 0.08 | 0.05 | 0.10 | 0.09 | 0.03 |
| r | 0.99 | 0.94 | 0.85 | 0.99 | 0.93 | 0.89 | 0.97 | 0.99 | 0.95 | 0.94 | 0.05 |
| R2 | 0.98 | 0.88 | 0.72 | 0.98 | 0.86 | 0.78 | 0.94 | 0.98 | 0.89 | 0.89 | 0.09 |
